# Experimental evidence on the role of framing, difficulty and domain-similarity in shaping behavioral spillovers

**DOI:** 10.1038/s41598-024-71988-x

**Published:** 2024-10-24

**Authors:** Anja Köbrich León, Julien Picard, Janosch Schobin

**Affiliations:** 1 Institute of Economics, University of Kassel, Kassel, Germany; 2 Department of Management, Economics and Industrial Engineering, Politecnico Di Milano, Milan, Italy

**Keywords:** Psychology and behaviour, Climate-change policy, Environmental economics

## Abstract

Does prompting people to volunteer for the climate spur or hamper further environmental engagement? We address this question in an online experiment with 10,670 German respondents. First, respondents read a text explaining how to help scientists fight climate change. Second, participants choose whether to do a real-effort task, like the behavior emphasized in the text. Third, respondents can sign a petition against climate change. In Study 1, we manipulate the narrative of the texts. We compare narratives condemning inaction or praising climate action against a neutral narrative (control) and an unrelated article (placebo). In Study 2, we investigate how the difficulty of the first behavior moderates behavioral spillovers. In Study 3, we test if the similarity between the domains of the two behaviors (e.g., environment, health) moderates spillover effects. None of our narratives increase the uptake of the real-effort task. Doing the real-effort task does not increase the likelihood of signing the petition either. Difficulty and domain-similarity do not moderate these effects.

*Protocol registration* The stage 1 protocol for this Registered Report was accepted in principle on January 1, 2023. The protocol, as accepted by the journal, can be found at: https://doi.org/10.17605/OSF.IO/JPT8G.

## Introduction

The IPCC report published in 2022 estimates that demand-side mitigation initiatives could reduce greenhouse-gas emissions by 40 to 70% by 2050, of which 5% could be achieved by behavioral change alone^1^. To achieve such cuts in emissions, one needs a widespread adoption of environmentally sustainable lifestyles. In this regards, a growing corpus of scientific evidence has studied how policies informed by behavioral insights can induce people to reduce their carbon footprint^2–5^. Yet, generalizing the adoption of environmentally sustainable lifestyles will also require people to act on their carbon handprint: their impact on others’ carbon footprints. Inducing people to mitigate their carbon-handprints will however take more than a nudge. Fortunately, environmental activists have not waited for scientists to advise them on the best method to start warning people about the urgency to act, although the narratives they have used to promote such actions have often been inconsistent^6–8^. Research on the so-called behavioral spillovers indicates that the framing of the arguments one uses to convince someone of the threat raised by climate change may spur or hinder further engagement in climate-friendly actions^9^. A better understanding of how the interaction between the phrasing of these arguments and the nature of the pro-environmental behavior promoted alter further engagement in environmentally sustainable actions is therefore needed.

This project explores how two narratives used by politicians, environmental activists or media outlets to promote environmental activism can foster or hinder further engagement beyond the behavior targeted. While the first narrative pushes us to act by condemning our inaction against climate change, the second narrative does so by praising being environmentally friendly. We expect that the first narrative increases engagement in the targeted pro-environmental behavior by triggering a will to tamper negative feelings (i.e., guilt from not doing enough), and the second increases the participation to the targeted behavior by triggering a will to conform with the pro-environmental identity it emphasizes. As such, we hypothesize that these narratives yield different behavioral spillover effects, as measured by respondents’ participation to another unrelated pro-environmental deed.

**Fig. 1:**
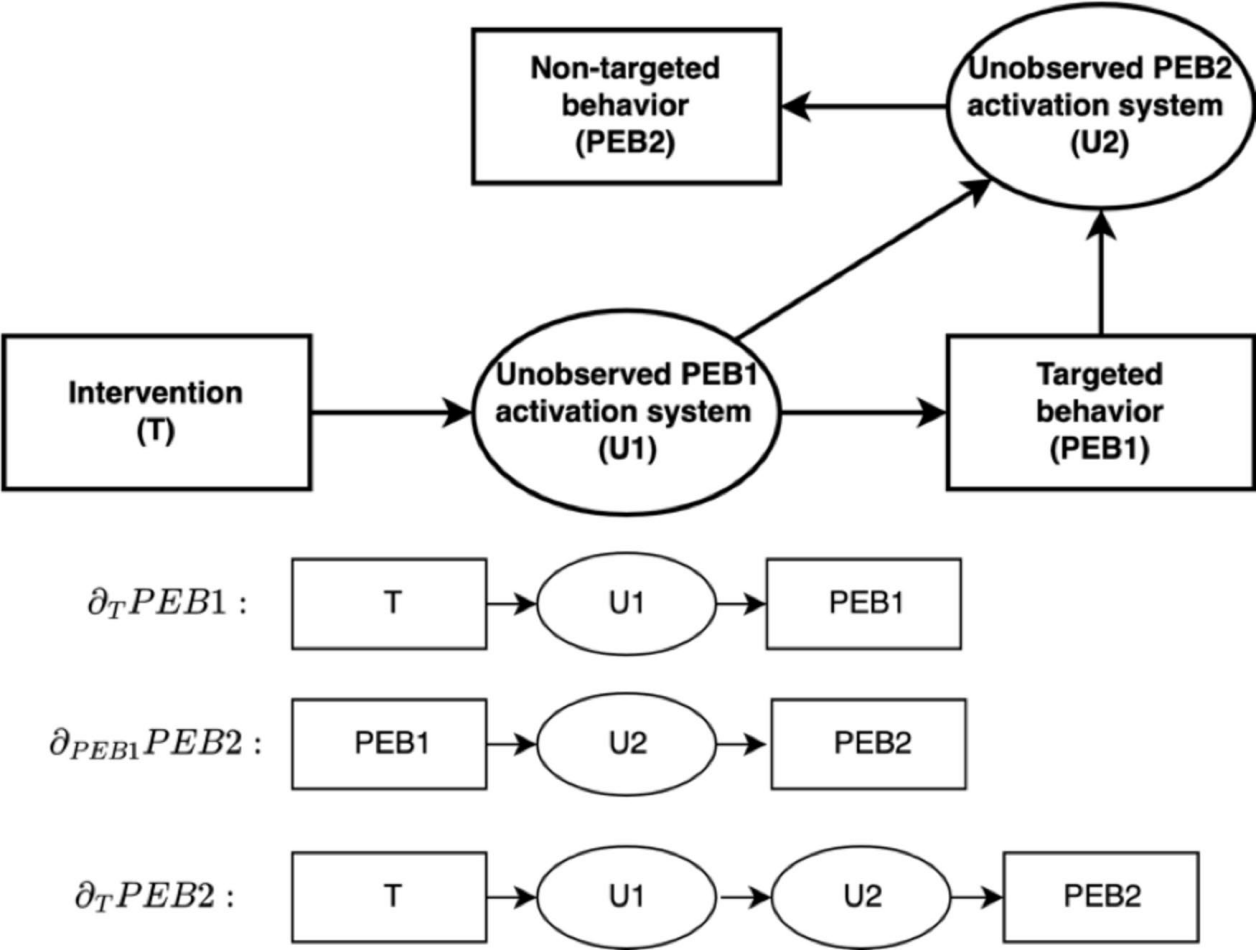
Behavioral spillovers.

We summarize the pathways through which we expect our narratives to trigger behavioral spillovers on a non-targeted behavior in Fig. 1. We denote by Δ_T_PEB2 the total spillover effect of narrative T on the non-targeted behavior. It corresponds to the definition of behavioral spillovers proposed by Galizzi and Whitmarsh^10^. In what follows, we refer to this effect as the *total spillover effect* of narrative T. This total spillover effect can be decomposed into two channels of influence of the narrative on the non-targeted behavior: the effect of a change in the targeted pro-environmental behavior on the non-targeted behavior, and the direct effect of the narrative on the non-targeted behavior. More formally (see section “sampling plan”):

$$ \underbrace {{\Delta_{T} PEB2}}_{Total \;spillover \;effect} = \underbrace {{\partial_{T} PEB1 \times \partial_{PEB1} PEB2}}_{Indirect \;spillover\; effect} + \underbrace {{\partial_{T} PEB2}}_{Direct \;spillover \;effect} $$

Here, ∂_PEB1_PEB2 is the effect of doing the targeted behavior on the second non-targeted behavior. It corresponds to the definition of *behavioral spillovers* proposed by Carrico et al.^11^ or Maki et al.^9^. In the formula above, this effect is scaled by ∂_T_PEB1, the total effect of the treatment on the respondents’ decision to do the targeted behavior*.* We refer to this effect as the *indirect spillover effect* of narrative T. Finally, ∂_T_PEB2 captures the direct effect of narrative T on the non-targeted behavior. It depends on *how* (i.e., through which cognitive process) the narrative induced the execution of the targeted behavior. We refer to it as the *direct spillover effect* of narrative T.

Our objectives are threefold. First, we seek to determine if the two narratives described above trigger different *direct spillover effects*. Second, we analyze whether the level of effort exerted when undertaking the first pro-environmental behavior moderates the size of the *indirect spillover effects*. Third, we investigate if *direct and indirect spillover effects* are restricted to behaviors belonging to the same domain (e.g., climate action) or can occur in other domains than the first behavior triggering them (e.g., health). For this purpose, we conducted three consecutive studies to test each of these points.

From the literature reviews conducted by Truelove et al.^12^, Dolan and Galizzi^13^ and Nilsson, Bergquist and Schultz^14^ and the meta-analysis of Maki et al.^9^, three limitations of the literature on behavioral spillovers stand out. First, factors altering spillovers are often studied in isolation of each other in separate experiments^14^. This limits the generalizability of the conclusions one can derive as studies are not always comparable. We address this point by investigating in the same experimental framework factors supposed to alter the sign of spillovers (the framing of narratives), and their magnitude (difficulty and domain similarity of behaviors). Second, experiments on spillover effects are often underpowered^9^. This issue questions the reproducibility of the results reported by these studies. We are voluntarily conservative to avoid this pitfall: we choose conservative expected effect sizes, and we base our power analysis on two-sided tests. Finally, there is not yet a consensual definition of behavioral spillovers, implying they have often been measured in an inconsistent way^9,12,13^. Indeed, experimental designs used by previous studies can be classified into broadly three categories, as illustrated in Appendix D in the Supplementary Information. The first category corresponds to experiments where, after being randomly exposed to an intervention or allocated to a control condition, respondents engage in two consecutive behaviors^11,15–17^. Such designs allow to estimate the *total spillover effect* of being exposed to a given treatment on the second behavior (Δ_T_PEB2) by comparing the group exposed to the intervention with the control group. It does not allow, however, to causally estimate the *behavioral spillover effect* of undertaking the first behavior on the second behavior (∂_PEB1_PEB2). The second category encompasses experiments with structures similar to those in the first category, but with an additional control condition where participants are not proposed to undertake the first behavior^18–20^. Such designs allow to estimate the effect of being offered to do the first behavior on the second one by comparing the treatment conditions with the group who was no proposed to do the first behavior^18,20^. Yet, causally assessing the *behavioral spillover effect* of doing the first behavior on the second behavior (∂_PEB1_PEB2) is, again, not permitted by such designs. Finally, the third category of experiments randomizes participants to either a group where they are forced to undertake the first behavior, or a group in which they are not offered to do it^21^. These designs allow to estimate the *behavioral spillover effect* of doing the first behavior on the second one (∂_PEB1_PEB2). Yet, for interpreting results in this way, one must assume that (not) being proposed to engage in the first behavior is equivalent to freely deciding (not) to do it.

In this paper, we use a similar design than Alacevich, Bonev and Söderberg^22^ and Picard and Banerjee^23^ to estimate the different effects outlined in our definition of behavioral spillovers. Our design follows the structure of experiments of the first category, except that participants are further randomized into a condition where doing the first behavior is unconsciously facilitated or not by a choice architecture nudge. The dummy variable capturing respondents’ allocation to the choice architecture nudge is used as an instrumental variable to causally estimate the *behavioral spillover effect* of doing the first behavior on the second behavior (∂_PEB1_PEB2). Doing so allows us to derive an unbiased estimate of the *direct spillover effect* of our randomized interventions on the second behavior (∂_T_PEB2). Our three studies are conducted with this design.

*Study 1* We aim to compare and map the *direct spillover effects* yielded by two ways of promoting a pro-environmental behavior: either condemning environmental inaction, or emphasizing the positive consequences of being pro-environmental. To do so, we design four different newspaper articles: the first encourages participation in the first pro-environmental behavior (PEB1) by emphasizing the positive impact of being pro-environmental (henceforth, win–win treatment); the second encourages participation in PEB1 by emphasizing the negative impact of individual climate inaction (henceforth, doom-and-gloom treatment); the third article simply covers PEB1 without encouraging readers to undertake it (henceforth, control); and the fourth article deals with an unrelated subject (henceforth, placebo). We design a between-subject experiment where participants are randomly shown one of the four articles before being proposed to undertake PEB1, then PEB2.

PEB1 consists in helping the research team develop an app to help people assess the environmental impact of their food choices. As such, PEB1 can be considered as a carbon handprint related behavior: participants do not mitigate their environmental impact but help mitigate that of others. It consists of classifying 30 food pictures by their meat content. Respondents’ answers will help us create a dataset to train an algorithm to predict the carbon footprint of food items. PEB2 consists in signing a petition to call for redesigning the German car tax by introducing a bonus-malus system that stronger considers the CO_2_ emissions throughout the purchase process. As such, this behavior is also carbon-handprint related. The petition is real.

We posit the win–win and the doom-and-gloom treatments to increase participation to PEB1.

1. Hypothesis 1: Reading the article with a win–win framing increases participation to PEB1 (∂_T_PEB1 > 0). Two-sided test, posited effect size, Cohen’s d = 0.3 (we adopt a more conservative estimate than the meta-analysis of Shipley and van Riper^24^ who find pride explains pro-environmental behaviors with an effect size of d = 0.345). *H0* = *Reading the article with a win–win framing does not increase participation to PEB1 compared to the placebo and the control condition.*
2. Hypothesis 2: Reading the article with a doom-and-gloom framing increases participation to PEB1 (∂_T_PEB1 > 0). Two-sided test, posited effect size d = 0.3 (we adopt a more conservative estimate than the meta-analysis of Shipley and van Riper^24^ who find guilt explains pro-environmental behaviors with an effect size of d = 0.539). *H0* = *Reading the article with a doom-and-gloom framing does not increase participation to PEB1 compared to the placebo and the control condition.*

While we expect that both narratives have a *positive indirect spillover* effect because doing PEB1 will increase participation in PEB2 (∂_PEB1_PEB2 > 0), we anticipate narratives to yield opposite *direct spillover* effects on PEB2. Indeed, in their theoretical framework, Truelove et al.^12^ hypothesize that enhancing a social role (e.g., being pro-environmental) is likely to trigger positive spillover effects by inducing people to conform with the role made salient^25–27^. We posit that triggering pride through positive arguments makes people’s pro-environmental identity salient. As such, we assume that the “win–win” narrative will trigger positive *direct spillover effects* (∂_T_PEB2 > 0). On the other hand, Truelove et al.^12^ hypothesize that behavioral interventions inducing people to act to tamper negative feelings are likely to trigger negative spillover effects^28,29^. Indeed, one of the underlying mechanisms assumed to underpin negative spillovers is moral licensing. Moral licensing describes the fact that people feel entitled to “ease-off” after doing a first morally virtuous behavior to repair a deprecated identity^28,30,31^. We expect that the doom-and-gloom narrative induces people to do the targeted behavior to reduce guilt associated with not doing enough for the environment, as such triggering negative *direct spillover effects* (∂_T_PEB2 < 0).

(3) Hypothesis 3: Both treatments affect the indirect spillover effect $\left({\partial }_{T}PEB1\times {\partial }_{PEB1}PEB2\right)$ compared to the control condition. Two-sided test, posited effect size d = 0.17. We derive this expectation from the meta-analysis of Maki et al.^9^ who find an effect size for behavioral spillovers of d = 0.17. Note that their definition of a *behavioral spillovers effect* diverges from our definition of an *indirect spillover effect*. To the extent of our knowledge, there are no prior studies that we can use to determine the expected effect size for the *indirect spillover effects* the way we define it. *H0: Both treatments do not increase the indirect spillover effect (*${\partial }_{T}PEB1\times {\partial }_{PEB1}PEB2)$* compared to placebo and the control condition.*
(4) Hypothesis 4: Reading the article with a win–win framing increases the direct spillover effect on PEB2 (i.e. ∂_T_PEB2 > 0). Two-sided test, posited effect size d = 0.2 (to the extent of our knowledge, there are no prior studies that we can use to determine the expected effect size for this effect, as such we use a conservative Cohen’s d of 0.2). *H0* = *Reading the article with a win–win framing does not increase the direct spillover (∂*_*T*_*PEB2) compared to the placebo and the control condition.*
(5) Hypothesis 5: Reading the article with a doom-and-gloom framing decreases the direct spillover on PEB2 (i.e. ∂_T_PEB2 < 0). Two-sided test, posited effect size d = 0.2 (to the extent of our knowledge, there are no prior studies that we can use to determine the expected effect size for this effect, as such we use a conservative Cohen’s d of 0.2). *H0* = *Reading the article with a doom-and-gloom framing does not decrease the direct spillover (∂*_*T*_*PEB2) compared to the placebo and the control condition.*

The categorical variable capturing respondents’ allocation to one of the two narratives, placebo or control constitutes our main independent variable. Participation to PEB1 were coded as a dummy or as a continuous variable based on the number of pictures participants classify before deciding to stop the task. We used them as outcome variables when investigating the main effect of our treatment interventions (Hypotheses 1, and 2 above). Participation to PEB2 is coded as a dummy. It is used as an outcome variable when investigating the effect of PEB1 on PEB2 (Hypothesis 3 above) and the effect of reading a newspaper article on PEB2 (Hypotheses 4, and 5 above).

*Study 2* In our second study, we focus on the treatment intervention (win–win or doom-and-gloom) which triggered the largest *indirect spillover effects* in absolute terms. We experimentally manipulate the difficulty of PEB1 by varying the number of categories participants have to sort food pictures in across two experimental conditions in a between-subject design*.* We investigate whether difficulty affects the magnitude and the direction of doing PEB1 on PEB2. Different theories are competing regarding the effects of manipulating the difficulty of the initial behavior on participation to PEB2. The foot-in-the-door theory^32^ suggests that performing a first easy task increases the probability of performing a second harder task. On the other hand, several studies^9,13,33^ suggest that performing a first harder task increases the probability of observing positive behavioral spillover effects on a second easier task. In a field experiment, Gneezy et al.^30^ find that pro-social behavior that is perceived as costly consistently generates positive spillover effects. The authors explain this effect by suggesting that pro-social behavior can be viewed as part of a self-identity that is reinforced by the costliness of the behavior. This explanation is however in contradiction with Diekmann and Preisendorfer^34^ who show that the effect of environmental attitudes on pro-environmental behaviors diminishes with increasing behavioral costs and perceived inconvenience of the environmental behavior. Their study would suggest that costlier behaviors reduce the likelihood to observe positive spillovers. We seek to test these competing hypotheses in our second study. We randomized respondents in two conditions: hard versus simple PEB1.

In this study, we tested the following hypothesis:

(6) Hypothesis 6: The difficulty of PEB1 affects the effect of doing PEB1 on PEB2 (∂_PEB1_PEB2). Therefore, we expect the indirect spillover effect $\left({\partial }_{T}PEB1\times {\partial }_{PEB1}PEB2\right)$ to be different depending on the version of PEB1 performed. Two-sided test, predicted effect size d = 0.17 (to the extent of our knowledge, there are no prior studies that we can use to determine the expected effect size for this effect). *H0* = *The difficulty of PEB1 does not affect the indirect spillover effect (*${\partial }_{T}PEB1\times {\partial }_{PEB1}PEB2$*.*

The categorical variable capturing respondents’ allocation to one of these two treatment arms (simple versus hard PEB1) constitutes our main independent variable. Participation to PEB2 is coded as a dummy. It is used as an outcome variable when investigating whether the indirect effect of our treatment interventions is affected by the difficulty of PEB1 (Hypothesis 6 above).

*Study 3* In our third study, we focus on the treatment intervention of Study 1 and the level of difficulty of PEB1 in Study 2 which yielded the largest *indirect and direct spillover* effects in absolute terms. We experimentally manipulate the framing of PEB2 to test whether behavioral spillovers only occur between behaviors belonging to the same domain (i.e., environment). Namely, participants were either allocated to a condition where PEB2 consists in signing a petition supporting political action against climate change (environmental domain) or a condition where PEB2 consists in signing a petition supporting policies to reduce individual social isolation tendencies and loneliness (personal health domain). Evidence suggests that behaviors requiring similar resources (e.g., in terms of money, time, place of performance) are correlated^35,36^. Margretts and Kashima^21^ shows that positive behavioral spillovers occur between behaviors requiring similar resources (i.e., money instead of time). Previous empirical studies examining behavioral consistency focus on the relationship between behaviors belonging to a similar domain, such as environmental behaviors^21,33,35,37–41^, charitable donations^42–47^ or health-related behaviors^48–50^. Less well understood, however, is whether behavioral spillovers occur across different domains. The literature often assumes behavioral spillovers to happen between two decisions that belong to the same domain^12,13^ (e.g., preserving the environment). Yet, empirical evidence is scarce and mixed, with studies suggesting cross-domains spillovers might exist^11,51,52^, and others supporting the hypothesis that spillover effects are more prevalent between choices in similar domains^53,54^. Testing this assumption is important to determine if our understanding of behavioral spillovers is the right one. If it were to be rejected, one would have to look at other mediators than those currently studied.

(7) Hypothesis 7: The effect of doing PEB1 on PEB2 (∂_PEB1_PEB2) is different for the environmental condition compared to the health condition. Therefore, we expect the indirect spillover effect ${(\partial }_{T}PEB1\times {\partial }_{PEB1}PEB2)$ to be different depending on the target behavior. Two-sided test, predicted effect size d = 0.17 (to the extent of our knowledge, there are no prior studies that we can use to determine the expected effect size for this effect). *H0* = *The indirect spillover effect is not significantly different for the environmental condition than for the health condition.*
(8) Hypothesis 8: The *direct spillover* effect of reading an article with the selected narrative on PEB2 (∂_T_PEB2) is different for the environmental condition than for the health condition. Two-sided test, predicted effect size d = 0.20 (to the extent of our knowledge, there are no prior studies that we can use to determine the expected effect size for this effect). *H0* = *The indirect spillover effect of reading an article with the selected narrative on PEB2 is not significantly different for the environmental condition compared than for the health condition.*

The categorical variable capturing respondents’ allocation to one of these two treatment arms (environmental versus health domain) constitutes our main independent variable. Participation to PEB2 is coded as a dummy. It is used as an outcome variable when investigating whether the framing of PEB2 alters the effect of doing PEB1 on PEB2 (Hypothesis 7 above), and the effect of reading the article with the selected narrative on PEB2 (Hypothesis 8 above).

A summary of the different hypotheses tested in the three studies can be found in the design table (Table 1).

**Table 1:** Design table.

Question	Hypothesis	Sampling plan (e.g. power analysis)	Analysis plan	Interpretation given to different outcomes
*Study 1* How do the framing of narratives direct behavioral spillovers?	*Hypothesis 1* Reading the article with a win–win framing increases participation to PEB1 *H0* = *Reading the article with a win–win framing does not increase participation to PEB1 compared to the placebo and the control condition* *Hypothesis 2* Reading the article with a doom-and-gloom framing increases participation to PEB1 *H0* = *Reading the article with a doom-and-gloom framing does not increase participation to PEB1 compared to the placebo and the control condition* *Hypothesis 3* Both treatments increase the *indirect spillover effect* compared to the control condition *H0: Both treatments do not increase the indirect spillover effect compared to the placebo and the control condition* *Hypothesis 4* Reading the article with a win–win framing increases the *direct spillover effect* on PEB2 *H0* = *Reading the article with a win–win framing does not increase the direct spillover* *Hypothesis 5* Reading the article with a doom-and-gloom framing decreases the *direct spillover* on PEB2 *H0* = *Reading the article with a doom-and-gloom framing does not decrease the direct spillover on PEB2*	N = 5000 (n = 1250 per treatment group, see “Sampling plan – power analysis” section)	*Hypothesis 1 & 2* Probability linear model: *PEB1*_*i*_ = *a* + b * T_*i*_ + *u*_i_ PEB1_i_: 1 if do PEB1, 0 otherwise T_i_: 1 if allocated to “win–win” (“doom-and-gloom”) narrative for hypothesis 1 (2), 0 if in the control group *Hypotheses 3, 4 & 5* 2SLS model and studentized bootstrapped 95%-CI: *PEB1*_i_ = *a* + b * IV_i_ + c * T_i_ + *u*_i_ *PEB2*_i_ = *a’* + b’ * PEB1_i_*’* + c’ * T_i_ + *v*_i_ *PEB2*_*i*_ = *g* + t*T_*i*_ + *e*_*i*_ PEB2_i_: 1 if do PEB2, 0 otherwise IV_i_: 1 if allocated to condition where access to PEB1 is easy, 0 if difficult PEB1_i_’: Predicted values from the first-stage regression	*Hypothesis 1 & 2* Having *b* significantly higher than zero would provide support for these hypotheses. We will reject them otherwise *Hypothesis 3* Having *t -c’* significantly higher than zero would provide support for this hypothesis. We will reject it otherwise *Hypothesis 4* Having *c’* significantly higher than zero would provide support for this hypothesis. We will reject it otherwise *Hypothesis 5* Having *c’* significantly lower than zero would provide support for this hypothesis. We will reject it otherwise
*Study 2* Does the difficulty of PEB1 alter behavioral spillovers?	*Hypothesis 6* The difficulty of PEB1 affects the effect of doing PEB1 on PEB2. Therefore we expect the indirect spillover effect to be different depending on the version of PEB1 performed *H0* = *The difficulty of PEB1 does not affect the indirect spillover effect*	N = 2500 for the additional two treatment groups (see “Sampling plan – power analysis” section)	*Hypotheses 6 & 7* 2SLS model: *PEB1*_i_ = *a*_*1*_ + *b*_*1*_ * IV_i_ + *c*_*1*_ * (IV * Diff)_*i*_ + *d*_*1*_ * T_i_ + *u*_*1*i_ (PEB1 * Diff)_i_ = *a*_*2*_ + *b*_*2*_ * IV_i_ + *c*_*2*_ * (IV * Diff)_*i*_ + *d*_*2*_ * T_i_ + *u*_*2*i_ *PEB2*_i_ = *a’* + b’ * PEB1’_i_ + c’ * (PEB1 * Diff)_*i*_ + d * T_i_ + *v*_i_ Diff_i_: 1 is PEB1 is made difficult, 0 otherwise	*Hypothesis 6* Having *c’* significantly different from zero would provide support for this hypothesis. We will reject it otherwise
*Study 3* Does closeness between PEB1 and PEB2 alter behavioral spillovers?	*Hypothesis 7* The effect of doing PEB1 on PEB2 is different for the environmental condition compared to the health condition. Therefore, we expect the indirect spillover effect to be different depending on the non-targeted behavior *H0* = *The indirect spillover effect is not significantly different between the environmental condition and the health condition* *Hypothesis 8* The *direct spillover effect* on PEB2 is different for the environmental condition compared to the health condition *H0* = *The direct spillover effect on PEB2 is not different in the environmental condition compared to the health condition*	n = 2500 for the additional two treatment groups (see “Sampling plan – power analysis” section)	*Hypotheses 7 & 8* 2SLS model: *PEB1*_i_ = *a*_*1*_ + *b*_*1*_ * IV_i_ + *c*_*1*_ * (IV * Health)_*i*_ + *d*_*1*_ * T_i_ + *e*_1_*(T_i_*Health_*i*_*)* + *u*_*1*i_ (PEB1 * Health)_i_ = *a*_*2*_ + *b*_*2*_ * IV_i_ + *c*_*2*_ * (IV * Health)_*i*_ + *d*_*2*_ * T_i_ + *e*_*2*_ * (T_*i*_ * Health_*i*_*)* + *u*_*2*i_ *PEB2*_i_ = *a’* + b’ * PEB1_i_ + c’ * (PEB1 * Health)_*i*_ + d * T_i_ + e * (T * Health)_*i*_ + *v*_i_ Health_i_: 1 is PEB2 is framed as a health-related behavior, 0 otherwise	*Hypothesis 7* Having *c’* significantly different from zero would provide support for this hypothesis. We will reject it otherwise *Hypothesis 8* Having *e’* significantly different from zero would provide support for this hypothesis. We will reject it otherwise

## Methods

### Ethics information

We have obtained the ethic approval of German Association for Experimental Economic Research e.V. (No. bh41mpGC). Additionally, we obtained an ethics approval from the London School of Economics (reference 133,444). All research is performed in accordance with the relevant guidelines and regulations of these institutions. We seek the informed consent of people participating to the proposed studies. At the beginning of each study, participants were informed that we investigate the role of science in climate change. They were fully debriefed at the end of the survey (see Appendix A in the Supplementary Information). Norstat, our panel provider, provides participants with a typical market-based participation fee which is determined by the length of the questionnaire. In our case, participants received 0.6€ for a 10-min survey.

### Pilot data

We piloted the online survey experiment with a subsample of the panel provider to check the effectiveness of the different interventions. Specifically, we focused on tuning the cognitive complexity of the first task (PEB1). PEB1 consisted in classifying food pictures in terms of their meat content. We expect that more categories to rank pictures in and more pictures to rank increase the level of cognitive effort. We measured respondents’ perceptions of the difficulty of PEB1 and their level of fatigue. Furthermore, we measured how cognitively demanding the task is in terms of error rates. We also analyzed which factors in the choice environment unconsciously affect participation to PEB1 (i.e., salience and placement of buttons, availability of an exit option).

We also assessed whether the narratives displayed to respondents trigger the expected emotional reactions (e.g., guilt, shame, pride etc., see paragraph “Manipulation check- Study 1” in subsection “Treatment intervention” of section “Design”). We adjusted the texts based on the results of the pilot sessions. The subsample was excluded from the sample obtained in the final online survey experiment.

### Design

The entire data collection process was planned to take 2 months from start to finish. We adopted a fractional between-subject factorial design. The experiments consisted of five main steps: attention checks, online survey, treatment intervention, manipulation check, PEBs. The attention checks and the online survey are identical for all manipulation approaches studied. Differences in the experimental procedure only arise from the treatment intervention onwards, depending on the type of manipulation approach analyzed.

#### Attention checks

Before starting the survey, subjects were asked two questions to assess whether they are attentive. Respondents failing to answer correctly these two questions were excluded from the analysis. The first attention check takes the form of a 5-Likert scale question such as “*People are very busy these days and many do not have time to follow what goes on in the government. We are testing whether people read questions. To show that you’ve read this much, answer both “extremely interested” and “very interested””*. The second attention check is a multiple-choice question such as “*Most modern theories of decision making recognize that decisions do not take place in a vacuum. Individual preferences and knowledge, along with situational variables can greatly impact the decision process. To demonstrate that you’ve read this much, just go ahead and select both red and green among the alternatives below.”*

#### Pre-treatment questionnaire

Before respondents are allocated to treatment arms, they answered questions about their sociodemographic background. We measured respondents’ geographical location of residence, gender, age, and education level. These variables were used as to describe and assess the representativeness of our sample. We also assessed respondents’ pro-environmental and altruistic orientation using the scale provided by Bouman et al.^55^. We also measured respondents’ political orientation using a 10-Likert left–right scale. Finally, we measured respondents’ proneness to guilt and shame using the scale developed by Cohen et al.^56^. As part of an exploratory analysis, we tested whether these variables moderate our treatment effects. The full experimental setup, including the pre-treatment questionnaire, treatment texts, tasks, and information about the role of science, can be found in Appendix A in the Supplementary Information.

#### Treatment intervention

The experiment is designed in three steps. First, respondents are presented with a newspaper article covering the importance of PEB1. Second, respondents can do PEB1. Third, respondents are offered to undertake PEB2. In Study 1, we manipulate the framing of the narrative of the newspaper articles. In Study 2, we manipulate the difficulty of PEB1. In Study 3, we manipulate the domain of PEB2.

PEB1 takes the form of a series of tasks in which respondents have to categorize food pictures into different categories (e.g., “contains ruminant meat”, “contains non-ruminant meat”, “contains fish” etc.). They have 10 s to determine which category a given picture is more likely to belong to. In total, they had 30 pictures to categorize. We explained to respondents in the control and the treatment groups that their participation to PEB1 will help us develop a dataset to train an algorithm to predict the carbon footprint of food items. The purpose of this algorithm is to help people gauge the environmental impact of their food choices. We did not explain the purpose of PEB1 to participants allocated to the placebo group. Food pictures were taken by participants in a previous experiment.

PEB2 consists in signing a petition to call for redesigning the German car tax by introducing a bonus-malus system that stronger considers the CO_2_ emissions throughout the purchase process. In Study 3, some participants were randomized to another treatment arm where the petition called instead for supporting policies to reduce individual loneliness.

We plan to address the petition to the Petitions Committee of the German Bundestag. In contrast to petitions organized under private law, petitions submitted to the Petitions Committee of the German Bundestag have a legal right to be processed. The environmental petition (PEB2a) and the petition on personal health care (PEB2b) are submitted by the authors by post as a single petition (Einzelpetition). The petitions consist of the petition description and an explanation of relevance. Upon submission, a list of signers were also added. Respondents within the study had the opportunity to subscribe to this list of signatories. To do so, they had to state their name and address, including signature, on the list. To ensure a simple handling, the list was included at the end of the online survey.

In measuring behavioral spillovers, we disentangled the effect of reading a newspaper article with a specific narrative on participation to PEB2 (∂_T_PEB2) from the effect of doing PEB1 on PEB2 (∂_PEB1_PEB2). We follow the methodology proposed by Picard and Banerjee^23^. We randomly facilitate or hamper participation to PEB1 using an unconscious default nudge. The exact form of this nudge was determined during the pre-tests. This nudge was then used as an instrumental variable to handle potential self-selection bias when assessing the effect of doing PEB1 on PEB2 (see “Analysis plan” section for more details).

To minimize the possibility that participants notice a link between doing PEB1 and PEB2, subjects performed two filler tasks in between. The first task consists in moving the pointers of sliders to an indicated graduation. The second task consist in sorting pictures in two categories (“big” or “small”). See Appendix A in the Supplementary Information for details. Separating the two behaviors in this way helped mitigate potential experimenter demand effects.

*Study 1: manipulation of the narratives*

Using a between-subject fractional factorial-design, participants were randomly assigned to one of four groups: two treatment groups, one control group and a placebo. More precisely, we considered the following four different treatment arms:

1. A placebo where respondents are presented with a newspaper article covering a subject unrelated to PEB1.
2. A control group where respondents are presented with a newspaper article covering PEB1 in a descriptive way.
3. A first treatment group—referred to as the win–win treatment. Respondents are presented with the same newspaper article than in the control group. A paragraph is added to encourage participation to PEB1 focusing the adoption of morally esteemed pro-environmental identities, using expressions such as “what about being environmentally friendly” or “you can play a role for saving the planet”.
4. A second treatment—referred to as the *doom-and-gloom* treatment. Respondents are presented with the same newspaper article than in the control group. A paragraph is added to encourage participation to PEB1 by condemning morally disreputable climate inaction, using expressions such as “change your lifestyles that destroy the planet” or “stop being inactive”.

**Fig. 2:**
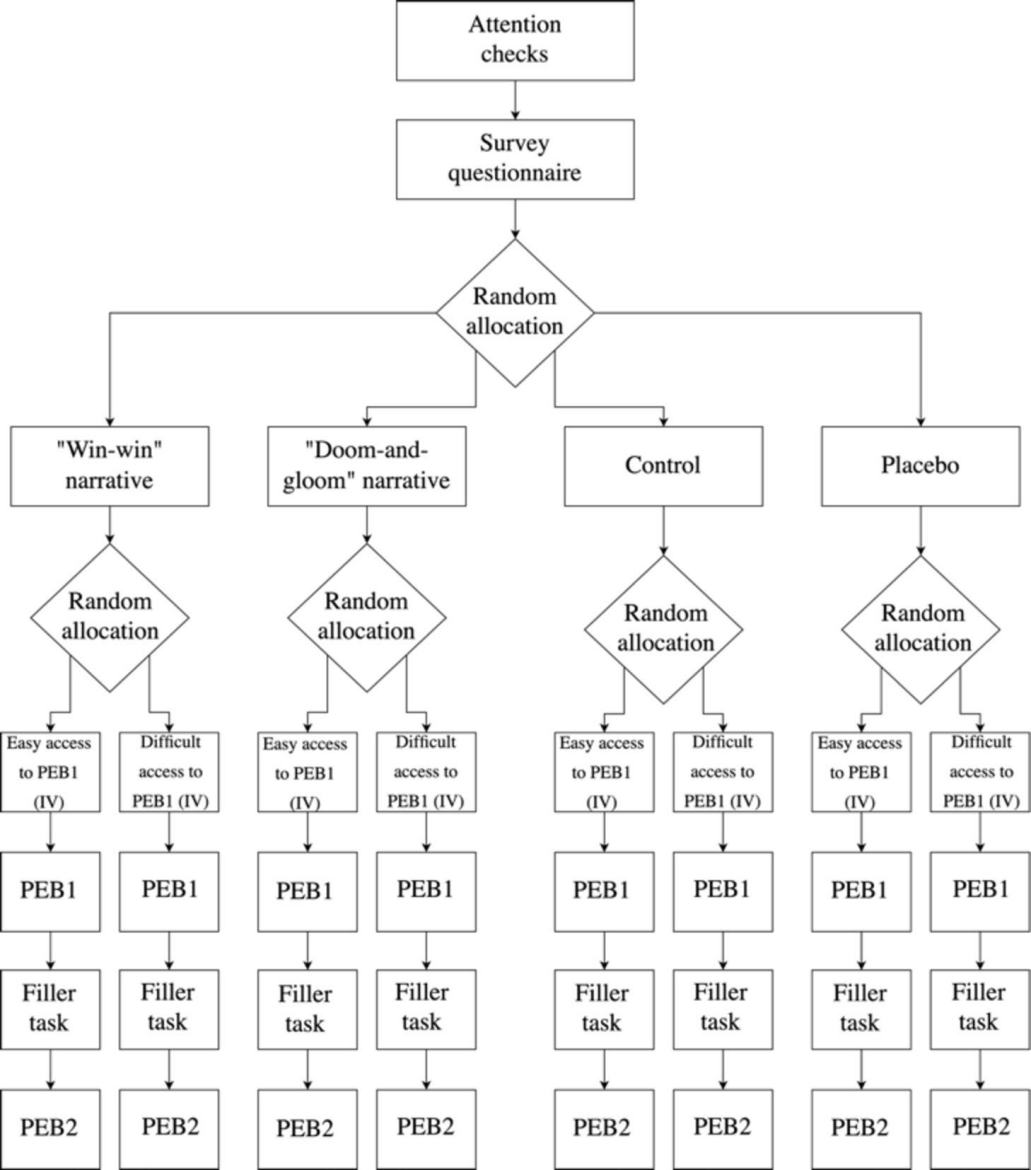
Experimental procedure for Study 1.

Respondents were asked to read the article they are allocated to before deciding whether to undertake PEB1. For the exact wording of the newspaper articles please refer to Appendix A in the Supplementary Information. The experimental design of Study 1 is depicted in Fig. 2.

Manipulation check—Study 1: after reading the newspaper article, participants answered a question to check if they read the article. This procedure allows us to check manipulation. We fine-tuned the phrasing of the article by adding questions in the pilot sessions on the feelings evoked by the treatments to assess their intensity. We expect the treatment effects to be moderated by respondents’ pro-environmental and altruistic values, and by their proneness to guilt, assessed in the pre-treatment questionnaire.

*Study 2: manipulation of the difficulty of PEB1*

Contingent on the outcome of the prior analysis, we chose the treatment group with the narrative yielding the strongest *indirect spillover* effect in absolute terms as intervention in the second manipulation approach. We allocated participants to one of the two following treatment arms:

1. In the *hard* condition, participants are allocated to the same version of PEB1 as in Study 1. Here participants are asked to categorize 30 pictures into 6 categories (“*Contains ruminant meat (e.g., lamb, beef)*”, “*Contains non-ruminant meat (e.g., pork, poultry)*”, “*Contains fish*”, “*Contains dairy (e.g., milk, cheese)*”, “*Contains eggs*”, “*Does not contain any animal-based product*”) within 10 s. See Appendix A in the Supplementary Information for visuals of the task.
2. In the *simple* condition, participants are allocated to a simpler version of PEB1. Here participants are asked to categorize 30 pictures into 2 categories (“*Contains animal-based products (e.g., meat, fish, dairy, eggs)*”, “*Does not contain animal-based products*”) within 10 s. See Appendix A for visuals of the task.

**Fig. 3:**
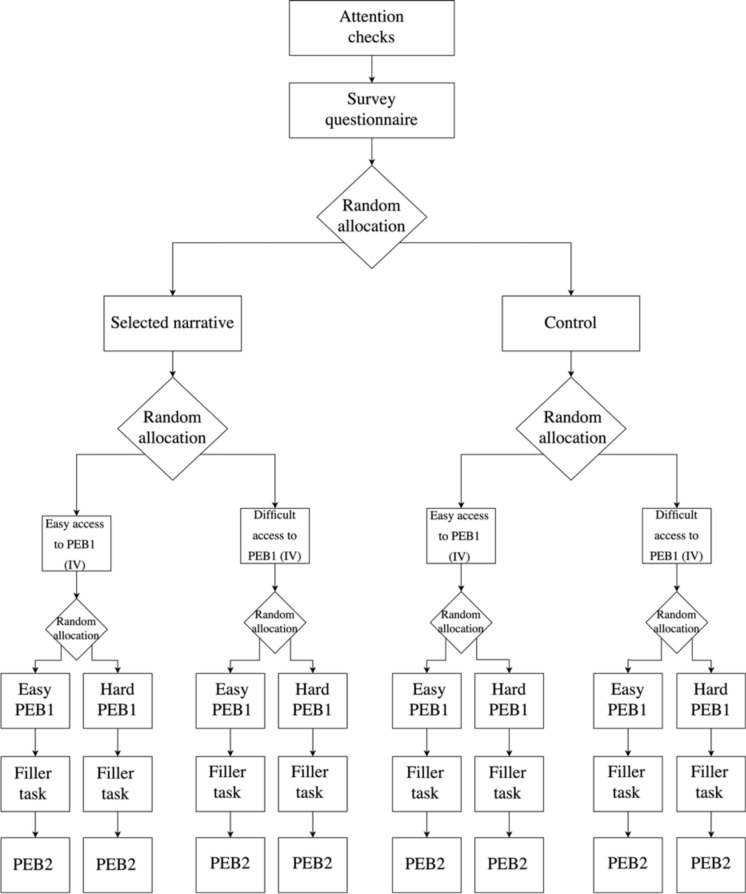
Experimental procedure for Study 2.

The duration and the number of categories were tested during the pre-test and the pilot sessions to make sure we successfully manipulate participants’ perception of the difficulty of PEB1. We initially planned to have 60 pictures to categorize within 5 s for each picture. The pre-tests revealed that 5 s was too short. We increased the time to 10 s. To keep the expected time spent in the experiment constant, we reduced the number of pictures to 30. Overall, the experimental design of Study 2 is depicted in Fig. 3.

Manipulation check—Study 2: to assess the objective difficulty of PEB1 we measured the time needed to classify the pictures in terms of their meat content. Furthermore, we asked participants in the pilot study to rate the perceived hardness of the task. Responses were collected on 10-point scale (ranging from “very hard” to “very easy”).

*Study 3: manipulation of the distance of the two behaviors*

Contingent on the outcome of the analyses of Study 1 and Study 2, the narrative and the level of difficulty of PEB1 yielding the largest *indirect and direct behavioral spillovers* in absolute terms were chosen in the third manipulation approach. In this study, we test the existence of cross-domain behavioral spillovers. To this end, participants were randomly assigned to two groups:

1. Environmental condition (PEB2a): participants were allocated to a condition where PEB2 consists in deciding whether to sign a petition supporting the redesign of the German car tax by introducing a bonus-malus system that stronger considers the CO_2_ emissions throughout the purchase process.
2. Health condition (PEB2b): participants were allocated to a condition where PEB2 consists in deciding whether to sign a petition in the area of loneliness prevention supporting policies to reduce individual loneliness or social isolation.

**Fig. 4:**
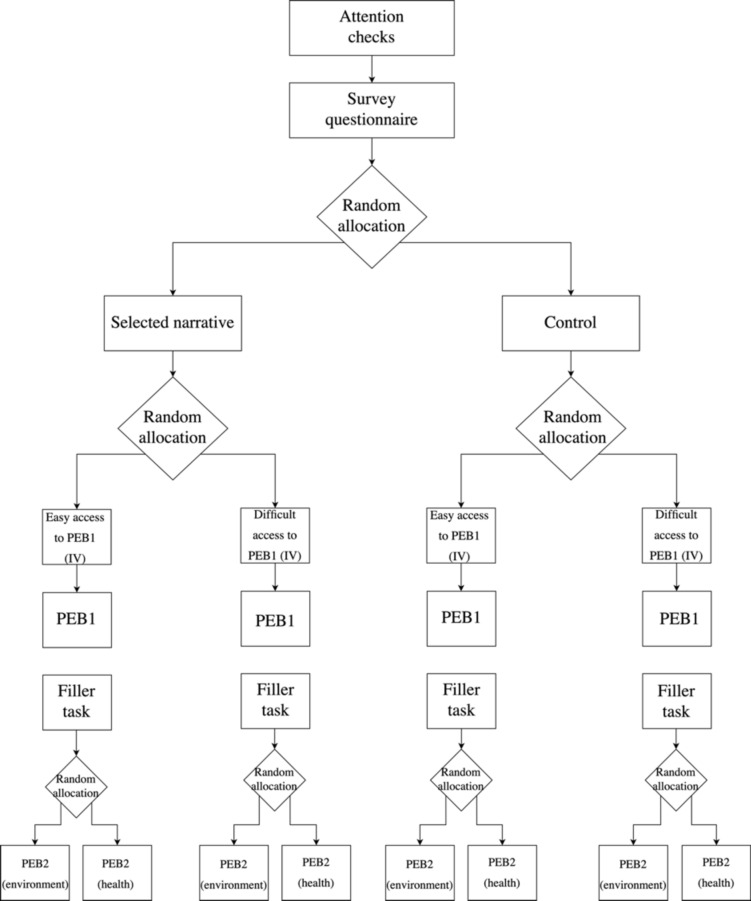
Experimental procedure for Study 3.

Overall, the experimental design of Study 3 is depicted in Fig. 4. See Appendix A for the exact wording of the petitions.

Manipulation check—Study 3: Participants answered questions measuring the perceived similarity of PEB1 and PEB2. We asked participants in the pilot study to rate the perceived similarity between PEB1 and PEB2. Responses were collected on 10-point scale (ranging from “very similar” to “very dissimilar”).

*Instrumental variable: manipulation of the choice architecture to facilitate the uptake of PEB1*

Across the three study, we randomized participants into two choice architecture nudges, facilitating or hardening the decision to undertake PEB1. Namely, when deciding whether to participate to PEB1, the salience of the “accept” button was varied. Similarly, when executing PEB1, the salience of the “withdraw” button was varied as well (see Appendix A for visuals of the choice architecture). When estimating the *indirect spillover* effect of doing PEB1 on PEB2, we instrumented the decision to do PEB1 by a dummy capturing respondents’ allocation to one of these two choice architectures. See section “sampling plan” for detailed explanation of the reasons to use such a strategy. See section “analysis plan” for more details on the estimation strategy.

#### Randomization

To assign individuals to treatments we employ pure randomization to achieve pre-treatment balance in treatment and control groups. The approach is reasonable. Previous studies show that the choice of the randomization method is not very important for the degree of balance of outcomes when samples are larger than 300 observations^57^.

#### Blinding

Data collection and analyses were performed blind to the conditions of the experiments. Researchers were provided with an anonymized dataset impeding them to know the identity of participants. Participation in PEB2a and PEB2b is different. To sign the petition, participants had to state their names and addresses, including signatures, at the end of the survey. However, they were not informed about the treatment group they had been allocated to. Those who chose to sign the petition were required to provide their names and addresses. The procedure corresponds to the submission of a single petition. Names and addresses were saved by the authors for the sole purpose of merging all signatures and were deleted after the petitions were sent by mail to the German Bundestag and the final data set for this experiment is assembled. There was no assignment by name between the participation in the petitions and the answers in the questionnaire.

### Sampling plan

#### Causal model of PEB-Spillovers

Figure 1 displays the causal paths leading an intervention to spill over a second non-targeted behavior, PEB2. Treatment interventions affect the PEB1 activation system U1, and therein simultaneously affects the decision to do PEB1 and the PEB2 activation system U2. In parallel, deciding whether to undertake PEB1 subsequently affect U2. The latter activation system determines the decision to do PEB2. More formally, we model the unobserved activation system U1 as a linear function of one’s exposure to the treatment intervention:

$$U1={\phi }_{1}\cdot Treatment$$

$\phi$ captures the strength of the influence of the treatment on activation system $U1$. The effort allocated to PEB1 is influenced by activation system $U1$, the intrinsic attractiveness of PEB1 $PEB{1}_{Atractiveness}$, and ancillary factors, in our case a choice architecture nudge denoted by $IV$:

$$PEB1=PEB{1}_{Atractiveness}+{\beta }_{U1}\cdot U1+{\beta }_{IV}\cdot IV$$

Here, ${\beta }_{U1}$ captures the strength of the influence of activation system $U1$ on the level of effort allocated to do PEB1. ${\beta }_{IV}$ captures the strength of the influence of the choice architecture nudge on the level of effort allocated to PEB1. Similarly, we assume the unobserved activation system U2 to be a linear combination of the influence of the activation system U1 and the level of effort allocated to PEB1:

$$U2={\phi }_{21}\cdot U1+{\phi }_{22}\cdot PEB1$$

${\phi }_{1}$ and ${\phi }_{2}$ capture respectively the strength of the influence of $U1$ and $PEB1$ on activation system $U2$. Activation system $U2$ is assumed to affect the level of effort allocated to do PEB2 in a linear way, as follows:

$$PEB2=PEB{2}_{Atractiveness}+{\beta }_{U2}\cdot U2$$

Here, $PEB{2}_{Atractiveness}$ captures the intrinsic attractiveness of PEB2. ${\beta }_{U2}$ captures the strength of the influence of the activation system $U2$ on the level of effort allocated to PEB2. The latter expression can be rewritten as follows:

$$PEB2=PEB{2}_{Atractiveness}+{\beta }_{U2}\cdot {\phi }_{22}\cdot PEB{1}_{Atractiveness}+({\beta }_{U2}\cdot {\phi }_{21}\cdot {\phi }_{1}+{{\beta }_{U2}\cdot {\phi }_{22}\cdot \beta }_{U1}\cdot {\phi }_{1})\cdot Treatment+{\beta }_{U2}\cdot {\phi }_{22}\cdot {\beta }_{IV}\cdot IV$$

We denote by ${\Delta }_{T}PEB2$, the variation in the level of effort allocated to PEB2 explained by one’s exposure to the treatment. We can show that:

$$ \underbrace {{\Delta_{T} PEB2}}_{Total \,spillover \,effect} = \underbrace {{\partial_{T} PEB1 \times \partial_{PEB1} PEB2}}_{Indirect\, spillover \,effect} + \underbrace {{\partial_{T} PEB2}}_{Direct\, spillover \,effect} $$

where ${\partial }_{T}PEB1\equiv \dot{{\beta }_{U1}}\cdot {\phi }_{1}$, ${\partial }_{PEB1}PEB2\equiv {\beta }_{U2}\cdot {\phi }_{22}$, and ${\partial }_{T}PEB2\equiv {\beta }_{U2}\cdot {\phi }_{21}\cdot {\phi }_{1}$. As such, treatment interventions affect the decision to do PEB2 through two pathways:

- An indirect spillover effect: the change in PEB1 – induced by the intervention – on PEB2 (∂_T_PEB1 × ∂_PEB1_PEB2). This effect does not depend on how the treatment affected the PEB2 activation system U2. As such, it should only capture the extent to which individuals perceive PEB1 and PEB2 as complementary (∂_PEB1_PEB2 > 0) or substitutable (∂_PEB1_PEB2 < 0).
- A direct spillover effect: the influence of the treatment on PEB2 through its effect on the PEB2 activation system (∂_T_PEB2). In other words, this effect captures the effect of the treatment on how individuals perceive PEB1 and PEB2. It can either reinforce (∂_T_PEB2 > 0) or weaken (∂_T_PEB2 < 0) the degree of complementarity between PEB1 and PEB2.

Because of endogeneity issues, we can hardly obtain an unbiased estimate of these two effects, unless the causal path from the U1 to PEB1 is interrupted. Yet, forcing PEB1 on some individuals—or not offering it to others—will not produce a valid estimate of the effect of doing PEB1 on PEB2 (∂_PEB1_PEB2). Forcing PEB1 may fundamentally change the nature of this behavior which may not produce the same effect on PEB2 than if it were voluntary. Conversely, not having had the choice to do PEB1 is not the same as not having engaged in it voluntarily.

**Fig. 5:**
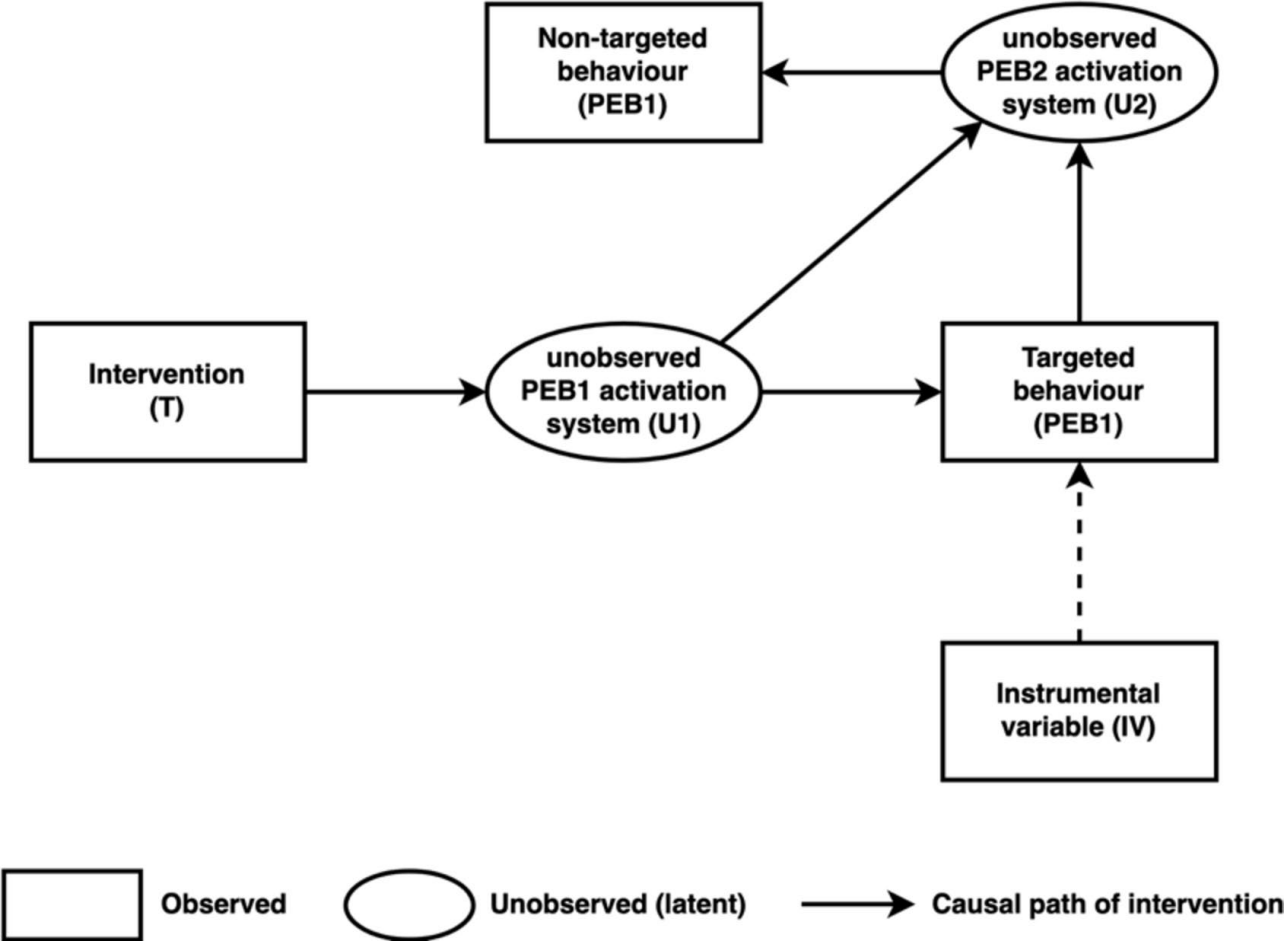
Causal DAG for experimental measurement of PEB-intervention spillover effects.

The only feasible option is therefore to use an instrumental variable approach^22,58^. In our case, we “nudge” individuals to do PEB1 in an additional randomized treatment by varying the choice architecture of the survey (see Appendix A for visuals). Contrarily to the framing of narratives we test in this experiment, choice architecture nudges lead to behavioral change by playing on people’s inattention. As such, it seems very unlikely that they would affect the unobserved activation system U1. This choice architecture nudge would therefore allow us to estimate the Complier Average Treatment Effect (CATE) of doing PEB1 on PEB2 (∂_PEB1_PEB2). The effect of such instrumental variable is shown in the causal DAG displayed in Fig. 5.

#### Simulation study to determine sample size

Based on the DAG in Fig. 5, we simulate data to estimate the necessary sample size to measure spillover effects. We want to achieve a power of 80% while keeping the Type I error rate at 5% (i.e. alpha ≤ 0.05).

The simulation is done in three steps. In the first step the data is sampled a thousand times for three scenarios with different sample sizes. The data generation process conforms to the causal hypotheses expressed in the DAG in Fig. 5. It is detailed hereafter. We set the parameters of the data generation process to obtain specific sample sizes (measured in Cohen’s d) for the main effect of the treatment on PEB1 (∂_T_PEB1), the total spillover effect of the treatment on PEB2 (Δ_T_PEB2), the direct spillover effect of the treatment on PEB2 (∂_T_PEB2), and the indirect spillover effect of the treatment on PEB2 (∂_T_PEB1 × ∂_PEB1_PEB2). The latter effect size is determined by subtracting the effect size of Δ_T_PEB2 with that of ∂_T_PEB2, using the fact that:

$${\partial }_{T}PEB1\times {\partial }_{PEB1}PEB2={\Delta }_{T}PEB2-{\partial }_{T}PEB2$$

In a second step, three effects are estimated from the simulated datasets: the total spillover effect of the treatment intervention on PEB2 (Δ_T_PEB2), the direct spillover effect of the treatment on PEB2 (∂_T_PEB2), and the indirect spillover effect of the treatment on PEB2 (∂_T_PEB1 × ∂_PEB1_PEB2). In estimating these effects, we follow the same estimation procedure than the one we used to analyze the experimental data (see section “analysis plan”). All calculations are done in R. We calculate the number of time we find statistically significant causal effects to estimate the statistical power one can reach with a given sample size, for a given scenario. In a final step, we analyze how sensitive the power is to three aspects of our experiment: (1) the strength of the IV in influencing the decision to do PEB1, (2) the baseline attractiveness of PEB1, and (3) the baseline attractiveness of PEB2.

#### Data generation process

The data is generated by a system of parametric equations that are consistent with the model outlined above. Treatment and the IV allocations amount to observing variables that take values of 0 or 1. Assuming a Bernoulli-distribution for this step is a contingent but natural assumption.

$$Treatmen{t}_{i}           Bernoulli\left(p=0.5\right)$$

$$I{V}_{i} Bernoulli\left(p=0.5\right)$$

$$\forall i\in \left[1,N\right]$$

We choose two statistical distributions to model the activation systems. We compare them for robustness checks. First, we assume the activation systems to be continuous and normally distributed. Furthermore, we assume that the decisions to undertake PEB1 and PEB2 are recorded as discrete binary choices. Since, in this case, we assume that we are not able to observe how inclined a participant is to engage in PEBs, we model the inclination to do PEBs as a continuous latent variable. We consider that it depends on two factors: the unobserved activation systems for PEBs, and the intrinsic attractiveness of PEB1 and PEB2, which can be considered as a fixed characteristic of the PEBs. In addition, we assume the inclination to do PEB1 to also depend on the nudge, $I{V}_{i}$.

*Version 1*

$$U{1}_{i} N\left(mean={\phi }_{1}\cdot Treatmen{t}_{i},\sigma ={\sigma }_{U1}\right)$$

$$U{2}_{i} N\left(mean={\phi }_{21}\cdot U{1}_{i}+{\phi }_{22}\cdot PEB{1}_{i},\sigma ={\sigma }_{U2}\right)$$

$$log\left(\frac{{p}_{i}^{PEB1}}{1-{p}_{i}^{PEB1}}\right)=PEB{1}_{Atractiveness}+{\beta }_{U1}\cdot U{1}_{i}+{\beta }_{IV}\cdot I{V}_{i}$$

$$PEB{1}_{i} Bernouilli\left(p={p}_{i}^{PEB1}\right)$$

$$Logit\left(\frac{{p}_{i}^{PEB2}}{1-{p}_{i}^{PEB2}}\right)=PEB{2}_{Atractiveness}+{\beta }_{U2}\cdot U{2}_{i}$$

$$PEB{2}_{i} Bernouilli\left(p={p}_{i}^{PEB2}\right)$$

$$\forall i\in \left[1,N\right]$$

${\sigma }_{U1}$ and ${\sigma }_{U1}$ are respectively the standard deviations of activation systems $U{1}_{i}$ and $U{2}_{i}$. $PEB{1}_{i}$ measures individual $i$’s decision to do PEB1.

We compare this to a second version, where we assume that PEB1 and PEB2 can be measured as observed continuous variables, to assess if and how the non-linearity of the first system affects the required sample size.

*Version 2*

$$U{1}_{i} N\left(mean={\phi }_{1}\cdot Treatmen{t}_{i},\sigma ={\sigma }_{U1}\right)$$

$$U{2}_{i} N\left(mean={\phi }_{21}\cdot U{1}_{i}+{\phi }_{22}\cdot PEB{1}_{i},\sigma ={\sigma }_{U2}\right)$$

$$PEB{1}_{i} N\left(mean=PEB{1}_{Atractiveness}+{\beta }_{U1}\cdot U{1}_{i}+{\beta }_{IV}\cdot I{V}_{i},\sigma ={\sigma }_{PEB1}\right)$$

$$PEB{2}_{i} N\left(mean=PEB{2}_{Atractiveness}+{\beta }_{U2}\cdot U{2}_{i},\sigma ={\sigma }_{PEB2}\right)$$

$$\forall i\in \left[1,N\right]$$

#### Simulation scenarios

Four estimates are of interest: (1) The total ATE for the Treatment on PEB1 (∂_T_PEB1), (2) the total ATE of the treatment on PEB2 (Δ_T_PEB2), (3) The indirect spillover effect of the treatment on PEB2 (∂_T_PEB1 × ∂_PEB1_PEB2) and (4) the direct spillover effect of the treatment on PEB2 (∂_T_PEB2). We consider three scenarios:

1. A favorable scenario: we hypothesize a large effect of the treatment on PEB1 (d ~ 0.88), a medium to large effect of the instrumental variable on PEB1 (d ~ 0.6), a medium to large total spillover effect (d ~ 0.7), a small to medium direct spillover effect (d ~ 0.4), and a small to medium indirect spillover effect (d ~ 0.3).
2. A middle scenario: we hypothesize a medium to large effect of the treatment on PEB1 (d ~ 0.58), a small to medium effect of the instrumental variable on PEB1 (d ~ 0.4), a small to medium total spillover effect (d ~ 0.37), a small direct spillover effect (d ~ 0.2), and a small indirect spillover effect (d ~ 0.17).
3. An adverse scenario: we hypothesize a small to medium effect of the treatment on PEB1 (d ~ 0.3), a small effect of the instrumental variable on PEB1 (d ~ 0.2), a small total spillover effect (d ~ 0.2), a small direct spillover effect (d ~ 0.11), and a small indirect spillover effect (d ~ 0.09).

First, we set the attractiveness parameters of the data generation process at plausible values. We assume they are not manipulated by the experiment. We pick the attractiveness of PEB1 and PEB2 (i.e., $PEB{1}_{Atractiveness}$ and $PEB{2}_{Atractiveness}$) to be -1.5 which corresponds to a base probability of about 18.2% to engage in the PEBs. In Version 2 both parameters are set to 0. Second, the autocorrelation parameter ${\phi }_{21}$ is set to $0.6$ (0.7 for Version 2), which corresponds to assuming a strong autocorrelation between the unobserved activation systems. Also, the variance (precision) parameters (σ_U1,_ σ_U2_) are fixed at 1 (for Version 2 additionally the precisions σ_PEB1,_ and σ_PEB2_ are set to 1).

After setting these “non-intervened” parameters we set the other parameters manually in a three-step process by repeatedly drawing a large sample (N = 5.000.000) from both data generation processes. Note that the choice of the expected effect sizes is constrained by the structure of the underlying causal model. In general, modifying any parameter will affect all effect size estimates, because they all impact the pooled variance estimate of PEB2. For this reason, certain scenarios are impossible, and others result from many parameter combinations. However, due to constraints on time and computing capacity we focus on just one set of parameters that produce the desired target scenarios (i.e. we assume that our power analyses are not sensitive to parameter changes in the class of parameters that yield identical Cohen’s d estimates). The three-step process to find a set of desired target parameters followed this procedure:

1. First, we fixed the strength of the “entry” and “exit” signal (i.e. ${\beta }_{U1}\wedge {\beta }_{U2}$ ) to achieve effect sizes for the total treatment effects (∂_T_PEB1 and Δ_T_PEB2) in each scenario that permit to achieve the targeted effect sizes for ∂_T_PEB2 and ∂_T_PEB1 × ∂_PEB1_PEB2.
2. Second, we iteratively adjusted ${\phi }_{1}$ and ${\beta }_{IV}$ to get the Cohen’s d of the three scenarios for ∂_T_PEB1 and ∂_IV_PEB1.
3. Third, we iteratively set ${\phi }_{22}$ to get the Cohen’s d of the three scenarios for ∂_T_PEB2 and ∂_T_PEB1 × ∂_PEB1_PEB2.

This three-step process is iterated itself until all Cohen’s ds correspond approximately (± 0.01) to the target scenario.

After setting all parameters, we sample repeatedly from the scenarios (Reps = 1000) for different sample sizes per treatment arm (N = 150, 200, 250, 350, 500, 750, 1000, 1250) and calculate the power as the fraction of significant results at alpha ≤ 0.05. The complete simulation code and all parameter values are included in the supplementary information.

#### Simulation results

**Fig. 6:**
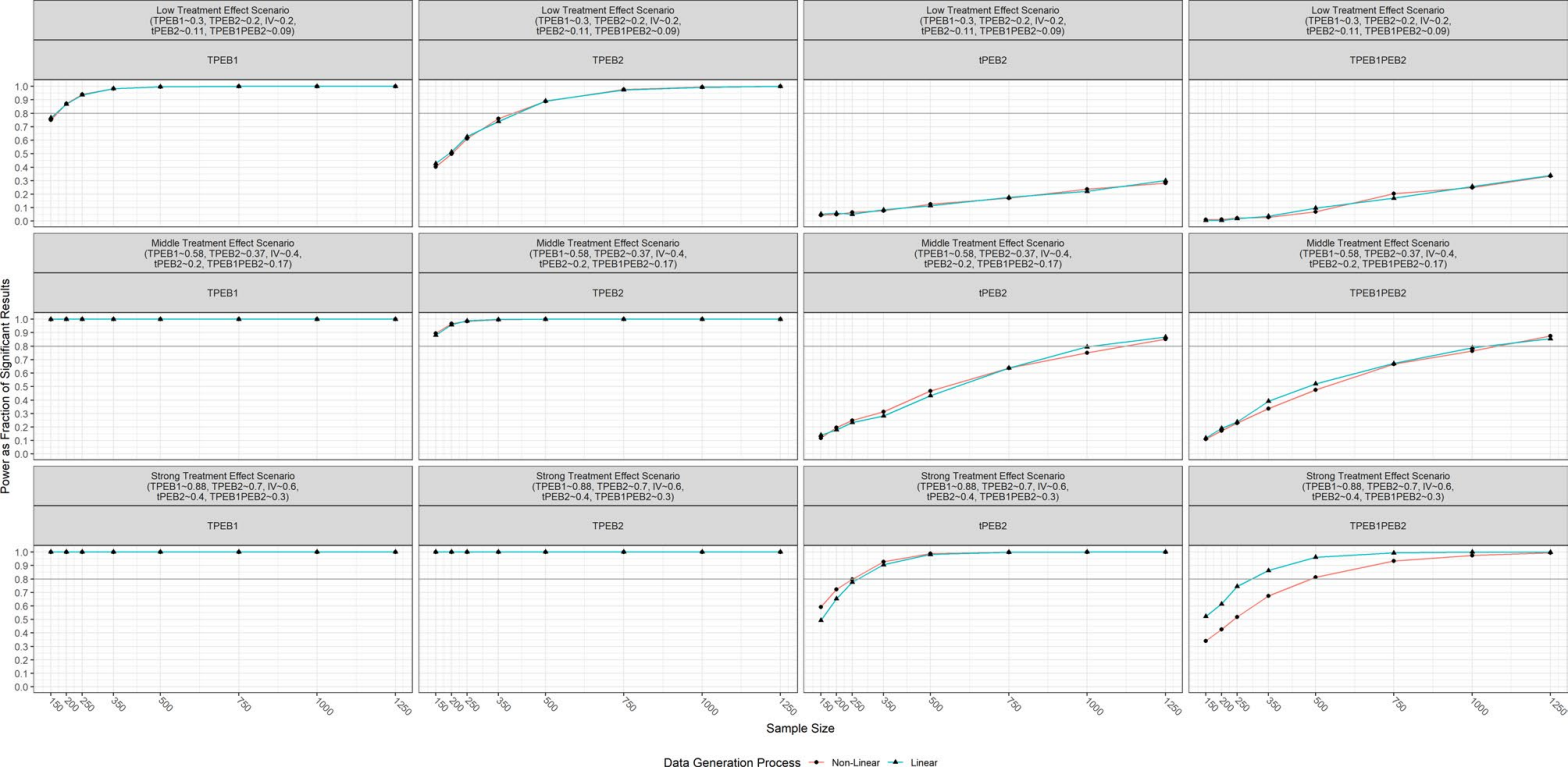
Stochastic simulation results of statistical power for three scenarios. Note: TPEB1 refers to the total effect of the treatment on PEB1 (∂_T_PEB1). TPEB2 refers to the total spillover effect (Δ_T_PEB2). tPEB2 refers to the direct spillover effect (∂_T_PEB2). TPEB1PEB2 refers to the indirect spillover effect (∂_T_PEB1 × ∂_PEB1_PEB2).

The results of the simulation are displayed in Fig. 6.

The main takeaways are the following:

1. Under the adverse scenario not even a sample size of N = 1250 per treatment arm would be enough to estimate the direct and indirect spillover effects (∂_T_PEB2 and ∂_T_PEB1 × ∂_PEB1_PEB2) with a power of 80% at α ≤ 0.05. A sample size of N = 500 per treatment arm would be enough to estimate ∂_T_PEB1 and Δ_T_PEB2 even in the adverse scenario.
2. Under the middle scenario, a sample size of N = 1250 per treatment arm is enough to estimate the direct and the indirect spillover effects (∂_T_PEB2 and ∂_T_PEB1 × ∂_PEB1_PEB2) with a power of 80%.
3. Under the favorable scenario N = 500 per treatment arm is enough to estimate the direct and indirect spillover effects (∂_T_PEB2 and ∂_T_PEB1 × ∂_PEB1_PEB2) with a power of 80%.

Note also that differences in the power are observable between the parametrizations, especially in the favorable scenario (Version1 vs. Version2). However, these differences are of minor practical relevance regarding the conclusions of the necessary sample size. This suggests that differences in the parametric form of the causal DAG should not impact the power too strongly, as long as the processes that are producing it are well behaved from a stochastic perspective (finite variances, not too skewed, etc.).

#### Power sensitivity analyses

Finally, we conduct three analyses to assess how sensitive the power of our design is to changes in: (1) the strength of the IV, (2) the attractiveness of PEB1, and (3) the attractiveness of PEB2. To this effect, we fix all parameters at the values of the middle scenario and vary the respective parameters for the impact of the IV (${\beta }_{IV}$), and the attractiveness of the PEBs ($PEB{1}_{Atractiveness}$ and $PEB{2}_{Atractiveness}$). We only conduct this experiment for Version 1 of the data generation process, because we already saw that differences between the two parameterizations are not too relevant. Furthermore, we only focus on the direct and the indirect spillover effects given that they are the estimates that consistently have the lowest power in our design.

**Fig. 7:**
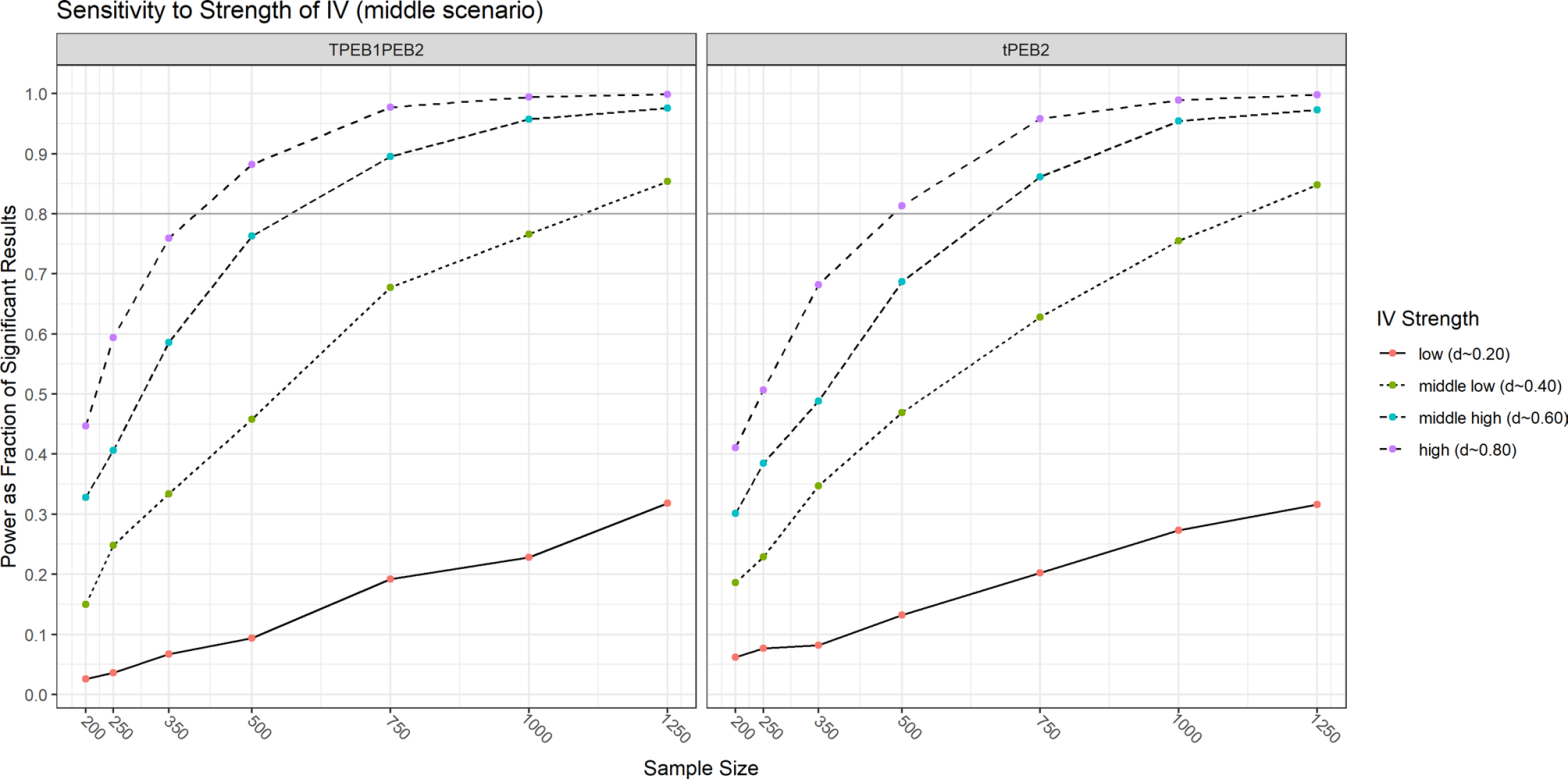
Sensitivity of power to strength of IV. Note: tPEB2 refers to the direct spillover effect (∂_T_PEB2). TPEB1PEB2 refers to the indirect spillover effect (∂_T_PEB1 × ∂_PEB1_PEB2).

The power of estimating the direct and the indirect spillover effects (∂_T_PEB2 and ∂_T_PEB1 × ∂_PEB1_PEB2) is very sensitive to the strength of the IV (see Fig. 7). While in the case of a low effect size IV (Cohen’s d ~ 0.2) not even a sample size of N = 1250 per treatment arm suffices to achieve a power of 80%, it is sufficient for a middle low strength of the IV (Cohen’s d ~ 0.4). The sample size requirements are substantially more moderate in the case of a middle to high effect size of the IV (Cohen’s d ~ 0.6, N ~ 750) or a very high strength of the IV (Cohen’s d ~ 0.8, N ~ 500). In conclusion, the pre-test strongly focused on the strength of the IV to gain statistical power.

**Fig. 8:**
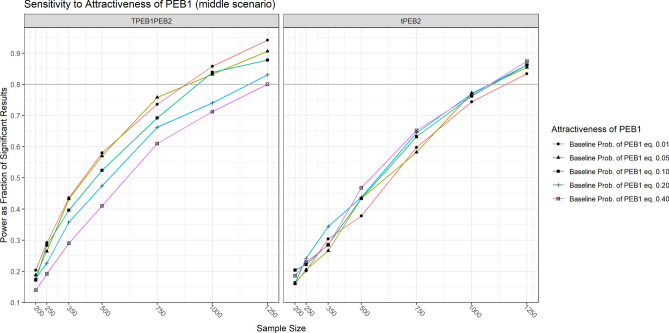
Sensitivity of power to attractiveness of PEB1. Note: tPEB2 refers to the direct spillover effect (∂_T_PEB2). TPEB1PEB2 refers to the indirect spillover effect (∂_T_PEB1 × ∂_PEB1_PEB2).

The power to estimate the indirect spillover effect is sensitive to the strength of the parameter capturing the attractiveness of PEB1 ($PEB{1}_{Atractiveness}$). We fix this parameter at five levels (-4.59512, -2.94439, -2.19725, -1.3862, -0.4045651) corresponding approximately to five base-probabilities (0.01, 0.05, 0.10, 0.20,0.40) of doing PEB1. The results suggest that there is a “sweet spot” somewhere between a base-probability of 0.01 and 0.10 that would substantially improve the power of our design (see Fig. 8).

**Fig. 9:**
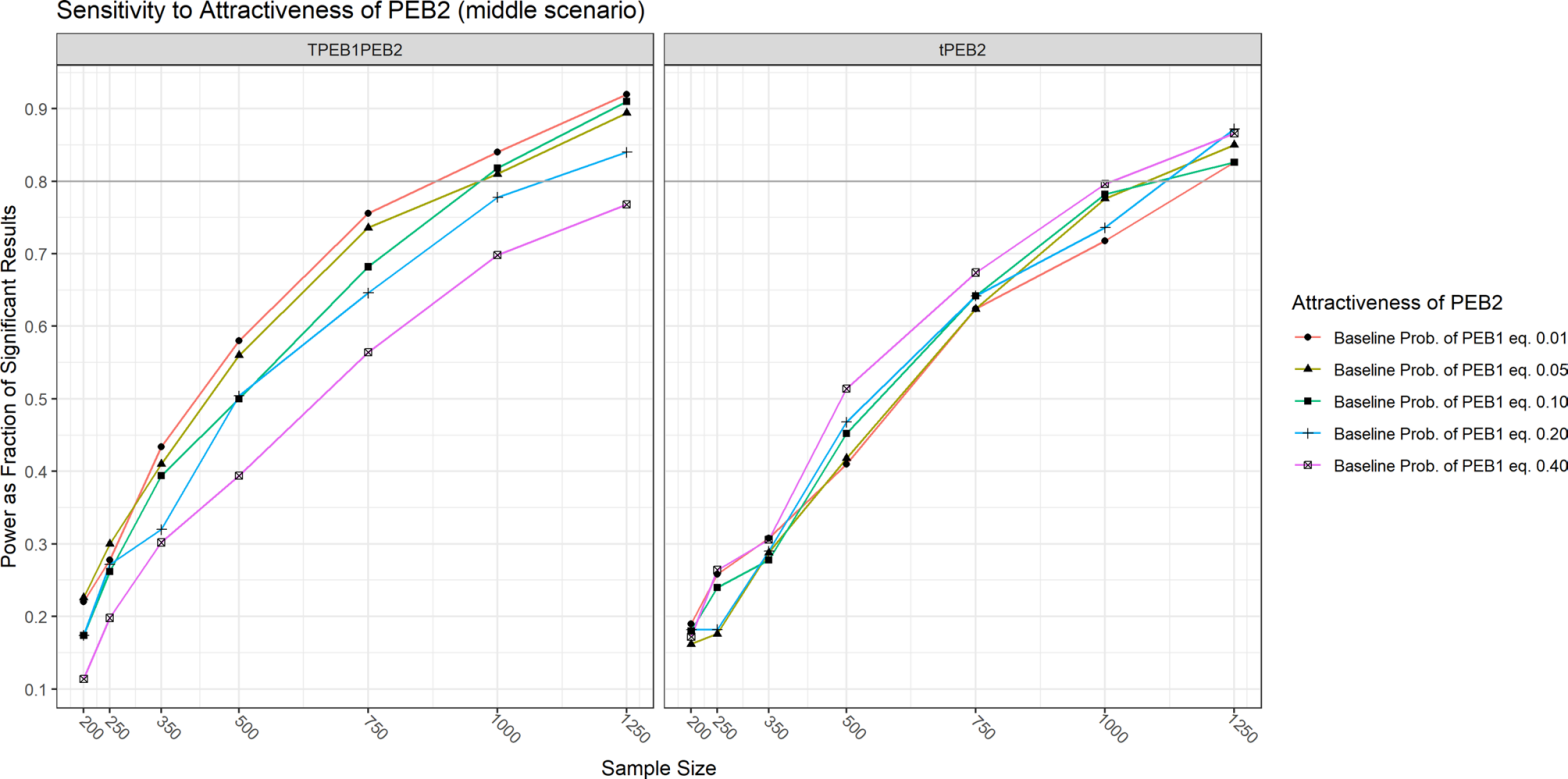
Sensitivity of power to attractiveness of PEB2. Note: tPEB2 refers to the direct spillover effect (∂_T_PEB2). TPEB1PEB2 refers to the indirect spillover effect (∂_T_PEB1 × ∂_PEB1_PEB2).

The power to estimate the indirect spillover effect is also sensitive to the strength of the parameter capturing the attractiveness of PEB2 ($PEB{2}_{Atractiveness}$), although to a lesser extent. We set this parameter at the same five levels than for PEB1 (-4.59512, -2.94439, -2.19725, -1.3862, -0.4045651). The results suggest that the power of our design increases if the base-probability of engaging in PEB2 is comprised between 0.01 and 0.10 (see Fig. 9).

In line with the results yielded by this power-analysis, we focused on designing the instrumental variable (i.e., the choice architecture nudge) to maximize our power during the pre-tests and the pilot sessions. Table 2 provides a summary of the sample sizes required for each study.

**Table 2:** Descriptive statistics by study.

	Study 1 (N = 5,492)	Study 2 (N = 2,622)	Study 3 (N = 2,556)	Total (N = 10,670)	*p*-value
Age					< 0.001
Median	45–54 yo (18.8%)	45–54 yo (23.3%)	45–54 yo (23.0%)	45–54 yo (20.9%)	
Min	18–24 yo (6.5%)	18–24 yo (4.6%)	18–24 yo (6.3%)	18–24 yo (6.0%)	
Max	> 65 yo (26.5%)	> 65 yo (18.1%)	> 65 yo (18.5%)	> 65 yo (22.5%)	
Income					< 0.001
Median	40 k-49 k€ (11.9%)	40 k-49 k€ (12.3%)	40 k-49 k€ (12.8%)	40 k-49 k€ (12.2%)	
Min	< 10 k€ (7.4%)	< 10 k€ (7.3%)	< 10 k€ (6.7%)	< 10 k€ (7.2%)	
Max	> 150 k€ (2.4%)	> 150 k€ (2.2%)	> 150 k€ (2.5%)	> 150 k€ (2.4%)	
Gender					0.208
Female	2782 (51.0%)	1282 (49.3%)	1262 (49.9%)	5326 (50.3%)	
Male	2612 (47.9%)	1282 (49.3%)	1224 (48.4%)	5118 (48.4%)	
Other	59 (1.1%)	34 (1.3%)	41 (1.6%)	134 (1.3%)	
Education					0.006
Median	Abitur or Eq. (24.1%)	Abitur or Eq. (24.5%)	Abitur or Eq. (24.6%)	Abitur or Eq. (24.3%)	
Min	No education (0.4%)	No education (0.4%)	No education (0.1%)	No education (0.4%)	
Max	PhD (1.6%)	PhD (2.1%)	PhD (1.7%)	PhD (1.7%)	
Political Belief					0.401
Mean	4.720	4.697	4.646	4.697	
Median	5 (31.5%)	5 (29.6%)	5 (27.6%)	5 (30.1%)	
Min	0 (2.7%)	0 (2.9%)	0 (2.8%)	0 (2.8%)	
Max	10 (2.7%)	10 (3.1%)	10 (2.3%)	10 (2.7%)	
SD	2.180	2.217	2.227	2.200	

We use a Wilcoxon test to check for differences in political beliefs across studies. We use a Chi-square test to check for gender differences. We use trend tests to check for differences in education, income, and age.

### Analysis plan

#### Statistical models

For Hypotheses 1, and 2, the dependent variable is either binary, and captures respondents’ decision to undertake PEB1, or continuous, and captures the number of pictures participants categorized before stopping doing PEB1. For Hypotheses 3, 4, 5, 6, 7, and 8 the dependent variable is binary and captures respondents’ decision to undertake PEB2. For Hypotheses 1, and 2 the main independent variables include the set of dummies capturing respondents’ allocations to our treatment interventions (newspaper articles).

In testing Hypotheses 1 and 2, we use linear models estimated by ordinary least squares as part of our main analysis such as:

1 $$PEB{1}_{i}=\alpha +\beta \cdot {T}_{i}+{\upsilon }_{i}$$

In testing Hypothesis 1, the dummy ${T}_{i}$ is equal to 1 if respondent $i$ is allocated to the win–win treatment group, zero if she is in the control group/placebo group. Symmetrically, ${T}_{i}$ is equal to 1 if respondent $i$ is allocated to the doom-and-gloom treatment group and zero if she is in the control group/placebo group when testing Hypothesis 2.

For testing Hypotheses 4 and 5, we use linear models estimated by two stage least-squares, each as:

1a $$ {\text{Stage 1}}:PEB1_{i} = \alpha + \beta \cdot IV_{i} + \delta \cdot T_{i} + \upsilon_{i} $$

2a $$ {\text{Stage}}\;2:PEB2_{i} = \alpha^{\prime} + \beta^{\prime} \cdot \widehat{PEB1}_{i} + \delta ^{\prime} \cdot T_{i} + \varepsilon_{i} $$

Here ${\delta }{\prime}$ is an estimate of the *direct spillover effect* $\left({\partial }_{T}PEB2\right)$ Again, for Hypothesis 4 the dummy ${T}_{i}$ is equal to 1 if respondent $i$ is allocated to the win–win treatment group, zero if she is in the control group/placebo group. Symmetrically, for Hypothesis 5 ${T}_{i}$ is equal to 1 if respondent $i$ is allocated to the doom-and-gloom treatment group and zero if she is in the control group/placebo group. $I{V}_{i}$ is equal to 1 when respondent $i$ is allocated to a condition where doing PEB1 is facilitated by a default nudge, 0 otherwise. ${\widehat{PEB1}}_{i}$ are the predicted values from regression (1a).

Hypothesis 3 is tested by estimating the additional regression:

3a $$ {\text{Stage}}\;3:PEB2_{i} = \gamma^{\prime} + \tau^{\prime}T_{i} + \varepsilon_{i} $$

Here, the *indirect spillover effect* (${\partial }_{T}PEB1\times {\partial }_{PEB1}PEB2$) is estimated as $\tau - \delta^{\prime }$. To decide on Hypothesis 3 we calculate a studentized bootstrapped 95% CI for $\tau - \delta^{\prime }$ based on 10.000 resamples of the original sample. If the 95% CI does not include 0, we considered the effect significant.

In testing hypotheses 6, we follow the same procedure as before: using linear models estimated by two-stage least squares, each as:

1b $$ {\text{Stage}}\;1:\left\{ {\begin{array}{*{20}c} {PEB1_{i} = \alpha + \beta_{1} IV_{i} + \beta_{2} \left( {IV_{i} \cdot difficult_{i} } \right) + \delta T_{i} + \upsilon_{1i} } \\ {(PEB1 \cdot difficult)_{i} = \tilde{\alpha } + \tilde{\beta }_{1} IV_{i} + \tilde{\beta }_{2} \left( {IV_{i} \cdot difficult_{i} } \right) + \tilde{\delta }T_{i} + \upsilon_{2i} } \\ \end{array} } \right. $$

2b $$ {\text{Stage}}\;2:PEB2_{i} = \alpha^{\prime} + \beta_{1}{\prime} \cdot \widehat{PEB1}_{i} + \beta_{2}{\prime} \cdot \widehat{{\left( {PEB1 \cdot difficult} \right)}}_{i} + \delta^{\prime } \cdot T_{i} + \varepsilon_{i} $$

Here, $difficul{t}_{i}$ is a dummy equal to 1 when respondent $i$ is allocated to the *hard* PEB1 condition.

Estimating coefficient ${\beta }_{2}{\prime}$ will allow us to test Hypothesis 6: whether the effect of doing PEB1 on PEB2 is mediated by the difficulty of PEB1, inducing a change in indirect spillover effect between the difficult and the easy PEB1 condition.

Finally, in testing Hypotheses 7 and 8, we use the following linear models estimated by two-stage least squares:

1c $$ {\text{Stage}}\;1:\left\{ {\begin{array}{*{20}c} {PEB1_{i} = \alpha + \beta_{1} IV_{i} + \beta_{2} \left( {IV_{i} \cdot health_{i} } \right) + \delta_{1} T_{i} + \delta_{2} \left( {T_{i} \cdot health_{i} } \right) + \upsilon_{1i} } \\ {\left( {PEB1 \cdot health} \right)_{i} = \tilde{\alpha } + \tilde{\beta }_{1} IV_{i} + \tilde{\beta }_{2} \left( {IV_{i} \cdot health_{i} } \right) + \tilde{\delta }_{1} T_{i} + \tilde{\delta }_{2} \left( {T_{i} \cdot health_{i} } \right) + \upsilon_{2i} } \\ \end{array} } \right. $$

2c $$ {\text{Stage}}\;2:PEB2_{i} = \alpha^{\prime} + \beta_{1}{\prime} \widehat{PEB1}_{i} + \beta^{\prime}_{2} \left( {\widehat{PEB1 \cdot health}} \right)_{i} + \delta_{1}{\prime} \cdot T_{i} + \delta_{2}{\prime} \cdot \left( {T \cdot health} \right)_{i} + \varepsilon_{i} $$

Here, $healt{h}_{i}$ is a dummy equal to 1 when respondent $i$ is allocated to the condition where PEB2 consists in signing a petition for supporting policies to reduce individual social isolation and loneliness.

Estimating coefficient ${\beta }_{2}{\prime}$ allows us to test Hypothesis 7: whether the effect of doing PEB1 on PEB2 is mediated by the framing of PEB2, inducing a change in indirect spillover effect between the environment and the health condition. Hypothesis 8 is tested by the significance of parameter ${\delta }_{2}{\prime}$ which estimates the difference in the direct treatment effect.

As part of an exploratory analysis, we investigated the heterogeneity of our treatment effects by interacting our moderators with the dummy capturing respondents’ allocation to the narratives (${T}_{i}$). More specifically, the moderating variables capturing pro-environmental and altruistic values were interacted with the dummy capturing allocation to the “win–win” narrative. The moderating variable capturing guilt proneness were interacted with the dummy capturing allocation to the “doom-and-gloom” narrative. This heterogeneity analysis was conducted for Hypotheses 1, 2, 4 and 5.

To construct the moderating variables capturing pro-environmental values, altruistic values and guilt proneness, we averaged the responses to the questions measuring each of these respective elements (see Appendix A).

As part of another exploratory analysis, we tested whether the mere exposure to information on PEB1 trigger spillover effects. As such, in doing so, we compared respondents allocated to the control group with those allocated to the placebo group. We used a statistical model similar to the one used for testing Hypotheses 3, 4, and 5.

For robustness checks, we run these different specifications by adding the covariates and social-demographic information measured in the survey questionnaire as controls and performed similar analyses adding previously excluded respondents to our sample (see section “sampling plan – data exclusion”). The use of linear models with dichotomous variables is valid as long as the predicted values of these models are bounded between 0 and 1, which is the case in our study. Yet, we run further robustness checks to ensure that our results are not an artefact of the statistical models chosen. Namely, we fitted probit models to estimate model (1). Further, we relied on the estimation procedure proposed by Rivers and Vuong^59^ and described by Wooldridge^60^ (Sect. “Econometric Analysis of Cross”) to estimate models (1’), (1’’) and (1’’’).^59^ We did not have to rely on maximum likelihood estimation as in Evans and Schwab^61^, and described by Wooldridge^60^ (Sect. “Panel Data”).

#### Inference criteria

We considered *p*-values from our linear regressions when drawing inferences. More precisely, we considered an effect size to be significant for *p*-values below 0.05. These p-values were corrected to account for multiple hypotheses problems by using Benjamini and Hochberg^62^ *p*-value correction procedure.

## Results

### Pilot study

We conducted a pilot study to refine the communicative strategies of environmental persuasion through newspaper articles. Data collection took place between August 2023 and October 2023. The study was designed to examine the psychological effects of different framing techniques on readers’ emotions and perceived task difficulty and similarity.

**Fig. 10:**
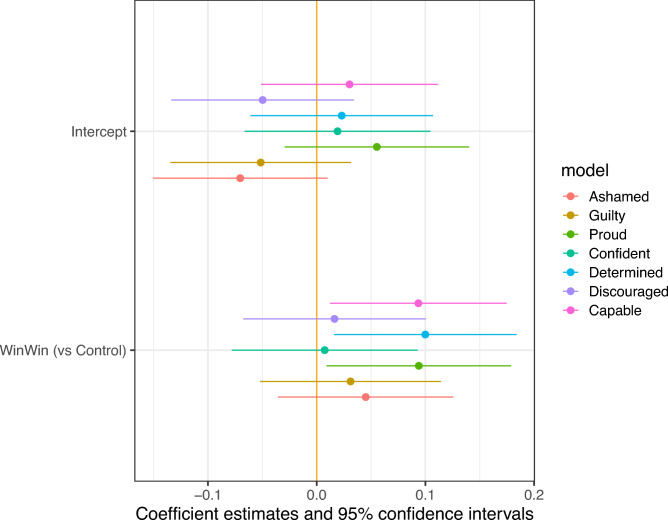
Emotions Associated with Win–Win Arguments.

**Fig. 11:**
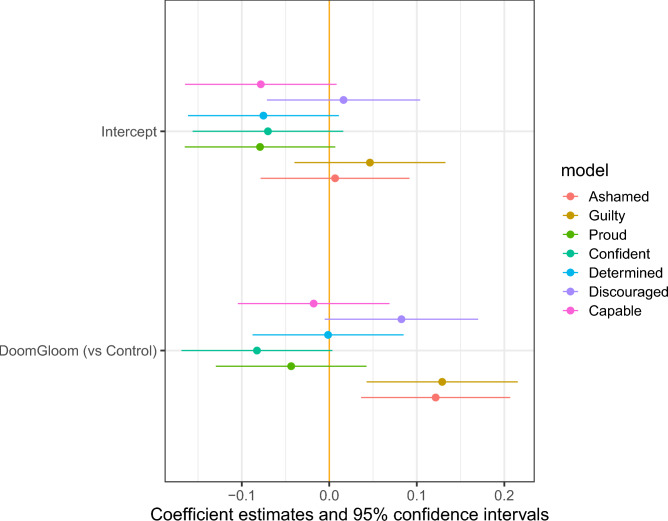
Emotions Associated with Doom-and-Gloom Arguments.

First, we tested the emotions evoked by reading each newspaper article. We looked at seven feelings and emotions (shame, guilt, pride, confidence, determination, discouragement, feeling capable). Figure 10 and 11 show coefficients from OLS regressions in which respondents’ reported emotions are regressed on their treatment assignments. There were 278 respondents in the control group, 265 in the win–win group, and 271 in the doom-and-gloom group. We find a clear difference in emotional responses, with the win–win text reinforcing positive feelings and the doom-and-gloom text reinforcing negative emotions. Compared to the control group, reading the win–win text increased feelings of ability, determination, and pride. Reading the doom-and-gloom text increased feelings of shame and guilt.

**Fig. 12:**
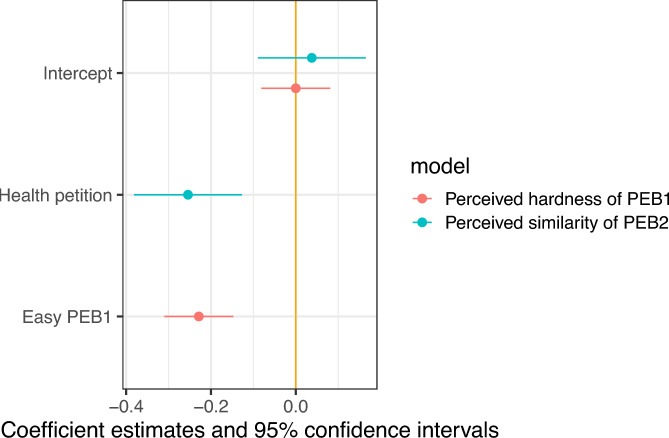
Perception that PEB1 is difficult and similar to PEB2.

Another dimension of our pilot focused on manipulating task difficulty in the context of PEB and the similarity between PEB1 and PEB2. In terms of difficulty, participants were randomly assigned to either a ‘hard’ or ‘easy’ condition in which they categorized a series of pictures. In the hard condition, participants categorized 30 pictures into 6 categories within 10 s. In the easy condition, participants categorized 30 images into 2 categories within 10 s. The graph in Fig. 12 shows the coefficients from OLS regressions regressing respondents’ perceptions of the difficulty of PEB1 on a dummy equal to one if they are in the easy condition, zero otherwise (red dots). There were 574 respondents in the hard version of PEB1 and 260 in the easy version. The figure shows that our task is perceived as less difficult when respondents are allocated to the easy condition.

To effectively test the manipulation of the domain of PEB2, which is necessary to detect cross-domain behavioral spillovers, we checked whether respondents perceived the environmental petition as similar to the real effort task (i.e., our targeted pro-environmental behavior) than the health-related petition, which was the case, as shown in Fig. 12. There were 703 respondents in the environmental version of PEB2 and 360 in the health version (blue dots). The graph shows respondents’ perception that PEB1 and PEB2 are similar, regressed on a dummy equal to one if respondents are in the health condition, zero otherwise.

### Sample characteristics

We collected data for the first study between 26th October 2023 and 8th January 2024. We partnered with Norstat, an online panel provider, for recruiting participants. The survey was designed on Qualtrics. Participants received a flat payment for doing the survey. Participation to our two behaviors (the real-effort task and the petition) was not incentivized, making these two tasks benevolent. In total, 7837 respondents took the survey, and 29.9% failed the attention checks, leaving us with a final sample of 5492 respondents. Following the criteria set in the registered report, we focused on the doom-and-gloom narrative and the control group in the next two studies. We collected data for the second study between 9th January 2024 and 6th February 2024. In total, 3724 respondents took the survey. We excluded 29.6% of participants as they did not pass the attention checks. This left us with a final sample size of 2622 respondents. Data for Study 3 was conducted simultaneously with Study 2. Overall, 3769 respondents took the survey, and 32.2% were excluded, leaving us with 2556 participants. The final sample comprises 10,670 observations, above our target of 10,000 respondents.

Table 2 shows descriptive statistics by study. The median respondent has a high school diploma or equivalent, earns between 40,000 and 49,999 euros per year, is between 45 and 54 years old, and is centrist on the political spectrum. Our sample is gender balanced (49.9% female, 48% male, 1.3% other).

**Fig. 13:**
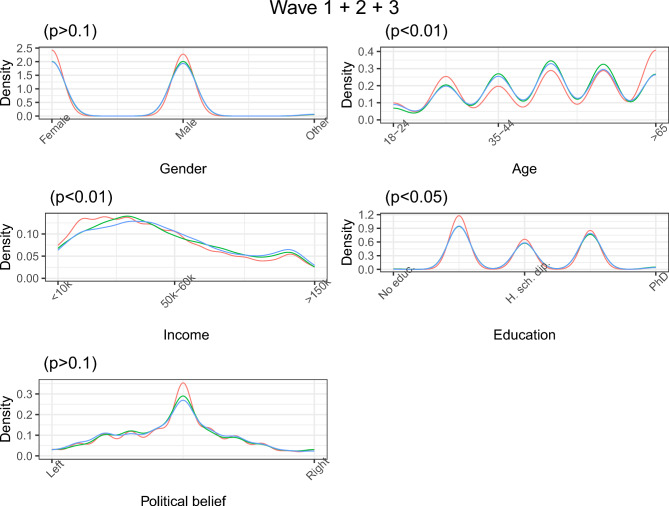
Distribution of covariates across studies.

Within each study, participants’ allocation to the treatment texts is randomized using Qualtrics’ randomization tool. We worked with Norstat, our panel data provider, to randomize respondents’ allocation to the studies. To check whether randomization worked, we tested for differences in gender, age, income, education and political beliefs between the treatment groups. Within each study, randomization was successful despite a small imbalance in income in Study 3 three. Being richer is positively correlated with being in the condition where the salience nudge hardens the uptake of PEB1 (Cohen’s d = 0.088, *p*-value = 0.023). We observe small statistical differences across studies. Participants in Study 1 are slightly poorer than participants in Studies 2 (d = − 0.081, *p* < 0.01) and 3 (d = − 0.129, *p* < 0.01). They are also slightly older than participants in Studies 2 (d = 0.053, p = 0.024) and 3 (d = 0.086, *p* < 0.01) and less educated than participants in studies two and three combined (d = − 0.062, *p* < 0.01). Participants of Study 2 are also slightly poorer than those of Study 3 (d = − 0.048, *p* = 0.094). Despite these statistically significant differences, Fig. 13 shows similar distributions of these covariates across studies.

**Fig. 14:**
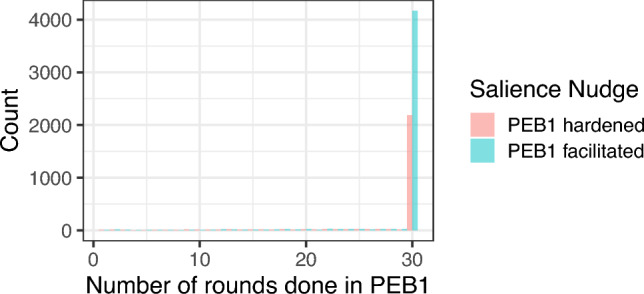
Histograms of the number of rounds done in PEB1.

**Fig. 15:**
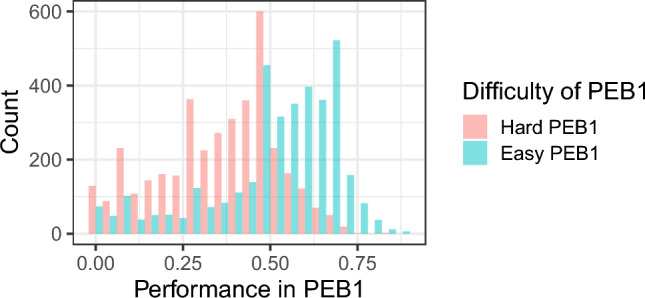
Histograms of performances in PEB1.

On average, around 70% of the full sample did the real-effort task. 85% of the participants who did the real effort task completed it by doing the 30 rounds (see Fig. 14). We observe more variations when looking at respondents’ performances during the real-effort task (see Fig. 15). We construct the performance score by averaging the correct rounds over 30. We deem an answer correct when it matches what was reported by respondents in a previous experiment. These respondents were followed over several months and reported the food they were eating as well as a picture of the food (that we used for the present experiment). Finally, on average, 29% of our sample signed the petition. We observe a large difference between the environment-related and the health-related petition, with 27% of signatories in the former case and 38% in the latter.

### Effect of treatment interventions on PEB1

Table 3 shows the average treatment effects of the intervention on participation in PEB1, the behavior aimed at supporting the research team in creating an app to assess the environmental impact of food choices. This is referred to as the carbon handprint behavior. It is measured either binary, capturing the respondent’s decision to perform PEB1, or continuous, capturing the number of pictures participants categorized before they stop performing PEB1. Columns one and two present the average treatment effect of being in a treatment or control condition on respondents’ participation in PEB1 compared to the placebo group. Columns three and four report the average treatment effect of providing neutral information relative to the placebo group. Columns five and six report the average treatment effect of win–win framing compared to the control condition. Columns seven and eight report the average treatment effect of doom-and-gloom framing compared to the control condition. We apply the Benjamini and Hochberg^62^ correction to conventional p-values.

**Table 3:** Effect of treatment texts on PEB1—Hypotheses 1 and 2.

Data included	Study 1		Study 1 without win–win & doom-and-gloom		Study 1 without placebo & doom-and-gloom				Study 1 without placebo & win–win	
Outcome	PEB1 (binary)	PEB1 (continuous)	PEB1 (binary)	PEB1 (continuous)	PEB1 (binary)	PEB1 (continuous)		PEB1 (binary)	PEB1 (continuous)	
	(1)	(2)	(3)	(4)	(5)	(6)		(7)	(8)	
Constant	0.503***	13.283***	0.491***	12.997***	0.525***		13.810***		0.518***	13.520***
	(0.013)	(0.389)	(0.015)	(0.440)	(0.016)		(0.452)		(0.016)	(0.449)
Salience nudge	0.389***	11.658***	0.414***	12.251***	0.367***		11.093***		0.381***	11.670***
	(0.011)	(0.333)	(0.015)	(0.460)	(0.016)		(0.478)		(0.016)	(0.471)
	p < 0.01	p < 0.01	p < 0.01	p < 0.01	p < 0.01		p < 0.01		p < 0.01	p < 0.01
	[0.477, 0.529]	[12.510, 14.034]	[0.461, 0.521]	[12.126, 13.850]	[0.494, 0.556]		[12.912, 14.682]		[0.487, 0.549]	[12.625, 14.384]
All vs. Placebo	− 0.004	− 0.022								
	(0.013)	(0.379)								
	p = 0.882	p = 0.988								
	[− 0.029, 0.021]	[− 0.762, 0.724]								
Control vs. Placebo			0.011	0.230						
			(0.016)	(0.463)						
			p = 0.732	p = 0.821						
			[− 0.020, 0.041]	[− 0.678, 1.134]						
Win–Win vs. Control					− 0.023		− 0.425			
					(0.016)		(0.475)			
					p = 0.371		p = 0.693			
					[− 0.054, 0.009]		[− 1.345, 0.518]			
Doom & Gloom vs. Control									− 0.024	− 0.367
									(0.016)	(0.472)
									p = 0.334	p = 0.702
									[− 0.055, 0.008]	[− 1.285, 0.564]
Number of observations	5492	5492	2757	2757	2727		2727		2760	2760
R2	0.178	0.1818	0.205	0.203	0.161		0.167		0.176	0.182
F-stat of Salience Nudge	1195.218	1222.825	720.309	709.462	515.097		538.467		576.188	615.208

This table displays the effect of the win–win, doom-and-gloom, and neutral information texts on respondents’ participation in the real effort task. Columns one and two present the average treatment effects (ATEs) of being in a treatment condition or the control group compared to the placebo group. Columns three and four present the ATEs of providing neutral information compared to the placebo group. Columns five and six present the ATEs of adding win–win arguments to neutral information. Columns seven and eight present the ATEs of adding doom-and-gloom arguments to neutral information. Robust standard errors are in parentheses. We apply Benjamini and Hochberg^62^ correction to conventional *p*-values (*p*). *p < 0.1, **p < 0.05, ***p < 0.01.

**Effect of the salient nudge**: Table 3 reveals a strong and significant effect of the salient nudge on respondents’ participation in the real effort task. Making it easier to participate in PEB1 increases the likelihood of doing it by 37.1 percentage points (*p* < 0.01) compared to making it harder to participate in PEB1. It also results in respondents categorizing 11 more pictures on average (*p* < 0.01). The F-statistic associated with the dummy that captures respondents’ allocation in the salience nudge is well above the convention threshold of 10. This indicates that our instrumental variable is strong^63–65^.

**Neutral information vs placebo:** Columns one and two of Table 3 present the results of an exploratory analysis in which we compare the placebo group with all other groups. We find no significant difference in participation in the real effort task. The third and fourth columns of Table 3 present the effect of providing neutral information on the decision to do PEB1 and the number of pictures categorized. This analysis is exploratory. Again, providing neutral information does not affect participation in PEB1 compared to the placebo group.

**Win–Win narrative vs neutral information**: The fifth and sixth columns of Table 3 present estimates of the effect of reading win–win arguments on the binary decision to do PEB1 and the number of pictures categorized. Emphasizing the benefits of action does not increase respondents’ participation in the real effort task compared to providing neutral information. There is no support for Hypothesis 1. This null result is not an artifact of our statistical method. It remains insignificant when using probit models or when controlling for social-demographic covariates and attitudinal information (see Table B.1 in Appendix B in the Supplementary Information). In Table C.1 in Appendix C in the Supplementary Information, we present the results of the exploratory analysis testing whether respondents’ altruism and pro-environmental attitudes mediate this effect. We find no evidence of heterogeneity in this result.

**Doom-and-Gloom narrative vs neutral information**: The seventh and the eighth columns of Table 3 present the effect of reading Doom-and-Gloom arguments on the decision to perform PEB1 and the number of pictures categorized. Emphasizing the cost of inaction does not increase respondents’ participation in the real effort task compared to providing neutral information. Thus, there is no support for Hypothesis 2. We even find a backfiring effect when pooling data from all studies together: reading doom-and gloom arguments slightly reduces participation in the real effort task (see Table B.2 in Appendix B). In Table C.1 in Appendix C, we present the results of the exploratory analysis that tests whether respondents’ guilt propensity mediates this effect. We find no evidence of heterogeneity in this result.

### Behavioral spillover effects on PEB2

Table 4 presents estimates of the effect of performing the real effort task on respondents’ likelihood of signing the environment-related petition (∂_PEB1_PEB2). In all columns, the variables capturing respondents’ participation in PEB1 are instrumented by the salient nudge. Columns one and two present the direct spillover effect of being in a treatment condition or the control group compared to the placebo group. Columns three and four present the direct spillover effect triggered by providing neutral information compared to the placebo group. Columns five and six present the direct spillover effects induced by win–win arguments compared to the control condition. Columns five and six present the direct spillover effects triggered by doom-and-gloom arguments compared to the control condition. We also report 95% confidence intervals of the indirect effect ∂_T_ PEB1 × ∂_PEB1_PEB2 estimated with bootstrap. Again, we apply the Benjamini and Hochberg^62^ correction to conventional p-values.

**Table 4:** Behavioral Spillover Effects on PEB2—Hypotheses 3, 4 and 5.

Data included	Study 1		Study 1 without win–win & doom-and-gloom		Study 1 without placebo & doom-and-gloom		Study 1 without placebo & win–win	
Outcome	PEB2	PEB2	PEB2	PEB2	PEB2	PEB2	PEB2	PEB2
PEB 1	Binary	Continuous	Binary	Continuous	Binary	Continuous	Binary	Continuous
	(19	(2)	(3)	(4)	(5)	(6)	(7)	(8)
Constant	0.252***	0.252***	0.270***	0.268***	0.300***	0.297***	0.298***	0.295***
	(0.024)	(0.022)	(0.030)	(0.028)	(0.036)	(0.033)	(0.034)	(0.031)
PEB1 (∂_PEB1_PEB2)	− 0.010	0.000	− 0.035	− 0.001	− 0.04	− 0.001	− 0.037	− 0.001
	(− 0.031)	(− 0.001)	(− 0.040)	(− 0.001)	(− 0.047)	(− 0.002)	(− 0.044)	(− 0.001)
	*p* = 0.882	*p* = 0.882	*p* = 0.693	*p* = 0.693	*p* = 0.693	*p* = 0.693	*p* = 0.693	*p* = 0.693
	[− 0.070, 0.050]	[− 0.002, 0.002]	[− 0.114, 0.044]	[− 0.004, 0.001]	[− 0.131, 0.051]	[− 0.004, 0.002]	[− 0.123, 0.050]	[− 0.004, 0.002]
All vs. Placebo (∂_T_PEB2)	0.024	0.024						
	(0.014)	(0.014)						
	*p* = 0.262	*p* = 0.262						
	[− 0.002, 0.051]	[− 0.002, 0.051]						
Control vs. Placebo (∂_T_PEB2)			0.027	0.027				
			(0.017)	(0.017)				
			*p* = 0.310	*p* = 0.310				
			[− 0.006, 0.060]	[− 0.006, 0.060]				
Win–Win vs. Control (∂_T_PEB2)					0.003	0.003		
					(0.017)	(0.017)		
					*p* = 0.940	*p* = 0.940		
					[− 0.031, 0.036]	[− 0.030, 0.037]		
Doom & Gloom vs. Control (∂_T_PEB2)							− 0.011	− 0.01
							(0.017)	(0.017)
							*p* = 0.779	*p* = 0.784
							[− 0.044, 0.023]	[− 0.043, 0.023]
Number of observations	5492	5492	2757	2757	2727	2727	2760	2760
95% bootstrapped CI of (∂_PEB1_PEB2 x ∂_T_PEB1)	[− 0.002, 0.002]	[− 0.001, 0.001]	[− 0.003, 0.002]	[− 0.002, 0.002]	[− 0.003, 0.004]	[− 0.003, 0.002]	[− 0.003, 0.004]	[− 0.003, 0.003]

This table displays the effect of doing the real effort task (instrumented by the salience nudge) on respondents’ likelihood of signing the environment-related petition. It also presents the effect of the win–win, doom-and-gloom, and neutral information texts on respondents’ likelihood of signing the petition. Columns one and two present the direct spillover effect of being in a treatment condition or the control group compared to the placebo group. Columns three and four present the direct spillover effect triggered by providing neutral information compared to the placebo group. Columns five and six present the direct spillover effects triggered by win–win arguments compared to the control condition. Columns seven and eight present the direct spillover effects triggered by doom-and-gloom arguments compared to the control condition. Robust standard errors are in parentheses. We apply Benjamini and Hochberg^62^ correction to conventional *p*-values (*p*). *p < 0.1, **p < 0.05, ***p < 0.01.

**Indirect Spillover Effect:** Participating in the real effort task does not causally increase respondents’ likelihood of signing the environment-related petition, despite positive correlations (see Table 5). As such, there is no support for Hypothesis 3. This null finding is not an artefact of the statistical method used. It remains non-significant when using Rivers and Vuong^59^‘s specification or when controlling for social-demographic covariates and attitudinal information (see Table B.3 Appendix B).

**Table 5:** Correlations between PEB1 and PEB2.

Data included	Study 1	
Outcome	PEB2 (binary)	PEB2 (continuous)
	(1)	(2)
Constant	0.241***	0.240***
	(0.010)	(0.010)
PEB1	0.032*	0.001**
	(0.013)	(0.000)
	*p* = 0.058	*p* = 0.020
	[0.007, 0.057]	[0.000, 0.002]
Number of observations	5492	5492
R2	0.001	0.001

This table displays the correlation between the decision to do PEB2 and the decision to do PEB1 (first column) and the number of rounds done in PEB1 (second column). We use OLS regressions. Robust standard errors are in parentheses. We apply Benjamini and Hochberg ^61^ correction to conventional *p*-values (*p*). *p < 0.1, **p < 0.05, ***p < 0.01.

**Direct Spillover Effect**: Table 4 also shows estimates of the direct effects triggered by our treatment texts on PEB2 (${\partial }_{T}PEB2$). We find no evidence that the Win–Win or Doom-and- Gloom treatments directly affected respondents’ likelihood of signing the environment-related petition. As such, there is no support for Hypotheses 4 and 5. This null finding is not an artefact of the statistical method used. It remains non-significant when controlling for social-demographic covariates and attitudinal information (see Table B.4 and Table B.5 in Appendix B). Further exploratory analyses suggest a negative direct effect of the win–win treatment for people being more altruistic than the median of respondents (see Table C.2 in Appendix C). However, this effect does not pass multiple hypothesis correction. We find no further heterogeneity.

The first two columns of Table 6 presents estimate of the effect of making PEB1 easier on the indirect behavioral spillover effect (∂_PEB1_PEB2). The variables capturing respondents’ participation in PEB1 are instrumented by the salient nudge. The variable Difficulty is a dummy equal to one when the difficulty of PEB1 is reduced, zero otherwise. The variable Health Petition is a dummy equal to one when the petition is about loneliness. Columns one and two present regressions where we interact the variable capturing participation in PEB1 with the difficulty of PEB1. Columns three and four present regressions where we interact the dummy capturing whether the petition is health-related with variables capturing participation in PEB1 and allocation to the doom-and-gloom treatment group.

**Table 6:** Effect of PEB1 difficulty and domain similarity—Hypotheses 6, 7 and 8.

Data included	Study 1 and Study 2		All studies without placebo & win–win	
Outcome	PEB2	PEB2	PEB2	PEB2
PEB1	Binary	Continuous	Binary	Continuous
	(1)	(2)	(3)	(4)
Constant	0.252***	0.252***	0.284***	0.282***
	(0.024)	(0.022)	(0.026)	(0.024)
Difficulty	0.002	0.002	0.005	0.006
	(0.042)	(0.040)	(0.012)	(0.012)
	*p* = 0.988	*p* = 0.988	*p* = 0.672	*p* = 0.625
	[− 0.081, 0.084]	[− 0.077, 0.081]	[− 0.019, 0.029]	[− 0.018, 0.030]
PEB1	− 0.010	0.000	− 0.023	− 0.001
	(0.031)	(0.001)	(0.033)	(0.001)
	*p* = 0.748	*p* = 0.748	*p* = 0.477	*p* = 0.477
	[− 0.070, 0.050]	[− 0.002, 0.002]	[− 0.088, 0.041]	[− 0.003, 0.001]
All vs. Placebo	0.024*	0.024*		
	(0.014)	(0.014)		
	*p* = 0.072	*p* = 0.071		
	[− 0.002, 0.051]	[− 0.002, 0.051]		
PEB1 x Difficulty	0.001	0.000		
	(0.058)	(0.002)		
	*p* = 0.988	*p* = 0.988		
	[− 0.112, 0.114]	[− 0.004, 0.004]		
Doom & Gloom vs. Control			− 0.001	− 0.001
			(0.012)	(0.012)
			*p* = 0.928	*p* = 0.935
			[− 0.025, 0.023]	[− 0.025, 0.023]
PEB1 x Petition			0.106	0.003
			(0.061)	(0.002)
			*p* = 0.262	*p* = 0.262
			[− 0.014, 0.226]	[0.000, 0.007]
Doom & Gloom vs. Control x Petition			0.042	0.041
			(0.023)	(0.023)
			*p* = 0.262	*p* = 0.262
			[− 0.003, 0.086]	[− 0.003, 0.086]
Health petition			0.016	0.020
			(0.047)	(0.044)
			*p* = 0.882	*p* = 0.843
			[− 0.075, 0.107]	[− 0.067, 0.107]
PEB1 x Health petition			0.106	0.003
			(0.061)	(0.002)
			*p* = 0.262	*p* = 0.262
			[− 0.014, 0.226]	[0.000, 0.007]
Doom & Gloom vs. Control x Health petition			0.042	0.041
			(0.023)	(0.023)
			*p* = 0.262	*p* = 0.262
			[− 0.003, 0.086]	[− 0.003, 0.086]
Number of observations	8112	8112	7936	7936

This table displays the effects of varying the difficulty of the real-effort task and the cause supported by the petition on direct and indirect spillover effects. In all columns, the variables capturing respondents’ participation in PEB1 are instrumented by the salient nudge. The variable “Difficulty” is a dummy equal to one when the difficulty of PEB1 is reduced, zero otherwise. The variable “Health Petition” is a dummy equal to one when the petition is about loneliness. Columns one and two present results from statistical model (2″). Columns three and four present results from statistical model (2′′′). Robust standard errors are in parentheses. We apply Benjamini and Hochberg^62^ correction to conventional *p*-values (*p*). *p < 0.1, **p < 0.05, ***p < 0.01.

**Difficulty of PEB1**: Table 6 shows that making PEB1 easier has not statistically significantly changed the indirect spillover effect. Therefore, there is no support for Hypothesis 6. Here again, the statistical method used does not influence our results (see Table B.6 in Appendix B).

**Similarity between PEB1 and PEB2:** We find suggestive evidence that the indirect spillover effect is higher with the health-related petition. We also find suggestive evidence that the direct spillover effect triggered by the Doom-and-Gloom narrative is higher with the health-related petition. Although these effects do not pass p-value correction, they are relatively robust to non-linear and linear specifications where controls are added (see Table B.7 in Appendix B).

**Table 7:** Exploratory analyses: Hypothesis 1 and 2 with performances as an outcome.

Data included	Study 1	Study 1 without win–win & doom-and-gloom	Study 1 without placebo & doom-and-gloom	Study 1 without placebo & win–win
Outcome	Performance in PEB1	Performance in PEB1	Performance in PEB1	Performance in PEB1
	(1)	(2)	(3)	(4)
Constant	0.180***	0.181***	0.167***	0.170***
	(0.007)	(0.007)	(0.007)	(0.006)
Salience nudge	0.144***	0.143***	0.153***	0.146***
	(0.008)	(0.008)	(0.008)	(0.006)
	*p* < 0.01	*p* < 0.01	*p* < 0.01	*p* < 0.01
	[0.129, 0.160]	[0.127, 0.158]	[0.138, 0.168]	[0.135, 0.157]
All vs. Placebo	0.006			
	(0.006)			
	*p* = 0.693			
	[− 0.006, 0.018]			
Control vs. Placebo		0.008		
		(0.008)		
		*p* = 0.589		
		[− 0.007, 0.023]		
Win–Win vs. Control			− 0.002	
			(0.008)	
			*p* = 0.928	
			[− 0.017, 0.014]	
Doom & Gloom vs. Control				− 0.006
				(0.008)
				*p* = 0.702
				[− 0.021, 0.009]
Number of observations	5492	2757	2727	2760
R2	0.136	0.152	0.125	0.136

This table displays the ATEs of win–win and doom-and-gloom arguments on respondents’ performances in the real effort task. We used OLS regressions where respondents’ performances are regressed on the dummy variables capturing their allocation to treatment texts. Robust standard errors are in parentheses. We apply Benjamini and Hochberg^62^ correction to conventional *p*-values (p). *p < 0.1, **p < 0.05, ***p < 0.01.

### Exploratory analyses

**Looking at performances**: We further explore whether our treatment interventions affect performances in the real effort task. We do not detect any significant effects of our win–win and doom-and-gloom arguments or neutral information (see Table 7).

In another exploratory analysis, we tested whether higher performances in the real effort task increased respondents’ likelihood of signing the petition. Results are presented in Table 8. To causally estimate the effect of higher performances on signing the petition, we instrumented performances by the difficulty of PEB1. The first column of Table 8 shows results of an OLS regression where performances in PEB1 are regressed on the difficulty of doing PEB1. The second column shows the results of a 2SLS regression where the perception of having made an effort for the environment is regressed on performances instrumented by the difficulty of PEB1. The third column shows the results of a 2SLS regression where the participation in PEB2 is regressed on performances instrumented by the difficulty of PEB1. Again, results in Table 8 reveal no evidence that doing well in the real effort task increased respondents’ likelihood of signing the petition.

**Table 8:** Exploratory analyses: Effect of performances in PEB1 on PEB2 and perception of effort.

Data included	Participants who did PEB1 in Study 1 and 2		
Outcome	Performance in PEB1	Perception of Effort	PEB2
	(1)	(2)	(3)
Constant	0.342***	2.127***	0.242***
	(0.006)	(0.079)	(0.033)
Difficulty	0.161***		
	(0.005)		
	*p* < 0.01		
	[0.150, 0.171]		
Performance in PEB1		0.881***	0.025
		(0.197)	(0.083)
		*p* < 0.01	*p* = 0.883
		[0.494, 1.267]	[− 0.138, 0.188]
Number of observations	5671	5671	5671
R2	0.164		

This table displays the ATE of varying the difficulty of the real-effort task on respondents’ performances (column one), the effect of performances (instrumented by the difficulty of PEB1) on respondents’ perception of having made an effort for the environment (column two), and the effect of performances (instrumented by the difficulty of PEB1) on respondents’ likelihood of signing the environment-related petition (column 3). We fitted an OLS regression for column one and 2SLS regressions for columns two and three. Robust standard errors are in parentheses. We apply Benjamini and Hochberg^62^ correction to conventional *p*-values (*p*). *p < 0.1, **p < 0.05, ***p < 0.01.

## Discussion

We contribute to a literature on gain and loss frames to foster pro-social behaviors. Evidence in the social psychology literature suggests that negative emotions or bad events are always more influential than positive emotions or good news^66,67^. Still, Homar and Kvelbar^68^ find that both strategies boost people’s pro-environmental intentions to act pro-environmentally^68^. Yet, this literature has focused mostly on stated preferences, using experiments with small sample sizes. Similarly, studies on the role of pride and guilt in inducing pro-environmental behaviors suffer from the same limitations^24^. We contribute to these strands of the literature by relying on a large sample size and a consequential real-effort task. Our findings align with recent meta-analyses indicating that information nudges are ineffective^2,69^.

We see five non-mutually exclusive explanations for our results. The first relates to participants’ attention. Respondents may not have read or processed the information presented in the articles. However, our data does not support this explanation. On average, 75% of the sample correctly answered our manipulation checks. Furthermore, our treatment effects are not mediated by the time spent reading the articles, whilst we would have expected that the shorter the time spent reading, the smaller the effect (see Table C.3 in Appendix C).

The second explanation regards heterogeneity. Our treatment interventions may have yielded opposite effects on different subsamples, which would average to zero on the full sample. Our exploratory analyses do not support this explanation either.

Third, we designed a consequential real-effort task. In that, we align with other experimental approaches seeking to move beyond pro-environmental intentions^70–72^. In our case, respondents’ payment was uncorrelated with the time spent doing the survey. In other words, they faced an opportunity cost from participating in the real-effort task. Previous studies stressing the benefit of acting or the costs of inaction looked at intentions^68^. Our results might thus indicate that such approaches are not powerful enough to translate people’s intentions into actions. This might be even more the case that most respondents did the real effort task. The remaining 30% that did not do it might be the hardest to convince. Furthermore, amongst those that did the task, only 15% did fewer than 30 rounds. This might not have provided enough variations to observe an effect at the intensive margin.

The fourth explanation concerns respondents’ trust in the source of information. Previous work shows that who delivers the information matters^73,74^. The success of win–win or doom-and-gloom arguments might depend on who initiates such attempts. It might have been that respondents exposed to these arguments sensed the experimenter was trying to steer them to do the real effort task by playing on their feelings. This could explain why we do not observe an effect of the win–win narrative and a (small) backfiring effect of the doom-and-gloom narrative. Similarly, not trusting the sender could have induced respondents not to believe that doing the real effort task would impact the environment. Yet, among respondents who did the task, those in the treated and control groups were more likely to feel they made an effort for the environment compared to the placebo group (see Table C.4 in Appendix C). This contradicts this explanation.

Fifth, the information treatment may have provided respondents with enough information to update their beliefs without significantly changing their intentions to act pro-environmentally. This is the case when respondents are motivated by things other than a genuine desire to solve environmental issues (e.g., warm glow, boost in self-esteem, social recognition). Our real effort task did not provide such contingent rewards. Contrary to other well-known pro-environmental behaviors, it was new to respondents. They could not have priors on whether doing it is a social norm or form any habits. Furthermore, respondents acted on their "carbon handprint" when doing the real effort task, i.e., others’ carbon footprint. The pathway through which it reduces carbon emissions is indirect. As such, doing it may not have yielded a strong feeling of achievement. In other words, it is possible that there were no "ropes" that our treatment interventions could use to spur participation.

This fifth explanation could also explain why we did not observe any indirect spillover effects. Contrary to other pro-environmental behaviors (e.g., recycling, eating vegetarian), our real effort task was not associated with values and social expectations. Its “artificial” nature might not have induced the processes leading to such spillover effects. This interpretation echoes recent experimental evidence suggesting that such consequential real-effort tasks often poorly correlate with people’s carbon footprint^75^.

Another explanation for spillover effects is that successfully doing first pro-environmental deeds motivates people to do more^76^. Here, our exploratory analysis seems to rule out this explanation. Higher performances increase the feeling that one has exerted an effort for the environment. Yet, it does not increase respondents’ likelihood of signing the environment-related petition (see Table 8).

Truelove et al.^12^ hypothesizes that the difficulty of the first action moderates behavioral spillover effects. We do not find evidence for this assumption. However, we only studied one dimension of behavioral difficulty. For instance, respondents might perceive some pro-environmental behaviors as difficult because doing them implies behaving against a social norm, irrespective of how easy it is to execute the behavior.

Finally, we find suggestive evidence of "cross-domain" spillover effects. However, our categorization of the two tasks in separate domains can differ from respondents’ perceptions. Indeed, the real effort task could be perceived as more pro-social than pro-environmental, given that respondents benevolently helped us encode food pictures. Similarly, the health-related petition about youth loneliness might have been perceived as more pro-social than the environment-related one, calling for a carbon tax on cars. Therefore, the pro-social nature of behaviors might be a stronger driver of spillover effects than their pro-environmental nature. These interpretations should be taken cautiously, as our results did not pass multiple hypothesis correction.

Yet, the idea that participants perceived the real effort task as more pro-social than pro-environmental could also explain some of our null effects. This would indeed explain why we do not observe any behavioral spillover effect when the petition is framed environmentally and suggestive positive spillover effects when it is framed with a health-related cause. This would also explain why our narratives did not affect at all participation in the targeted pro-environmental behavior. Indeed, our treatment texts contain arguments related to climate change and the consequences of action or inaction. Maybe emphasizing the mere warm glow of giving a hand to scientists, irrespective of the environmental cause, would have been more effective in increasing participation in the real-effort task.

## Conclusion

Providing information as a standalone intervention or coupling it with doom-and-gloom or win–win arguments does not foster pro-environmental action. Despite positive correlations, we also do not find causal evidence of behavioral spillover effects. In other words, doing the first pro-environmental action did not alter respondents’ likelihood to sign an environment- related petition, irrespective of the difficulty of the first action. Finally, we find suggestive evidence of cross-domain spillovers, with the caveat that this result does not pass multiple hypothesis correction.

Two main implications can be derived from our findings. First, our experiment questions the effectiveness of communication campaigns relying on information provision, alarmist warnings, or blissful optimistic messages. Second, our findings suggest that doing a pro-environmental action does not necessarily trigger behavioral spillover effects.

We see three explanations for these conclusions. First, we relied on a real effort task to proxy pro-environmental action instead of measuring intentions. Our treatment interventions might only shift people’s intentions without altering their behaviors. This would explain our inability to replicate previous findings. Second, our setting was peculiar as the real effort task was new to participants. People did not know if doing it was socially desirable or did not form any habits. The only reward associated with doing it is the mere knowledge that one made an effort for the environment. This might not be enough to motivate people to act. This may explain why we did not observe any first- and second-order effects. Third, participants could have perceived the targeted action as pro-social rather than pro-environmental. This would explain why we do not observe an effect of our interventions, as they were focusing on moral arguments associated to climate change rather than pro-sociality in general. It also explains why we do not observe any spillover effects of this task to the environmental petition.

These explanations imply different recommendations. Whilst the first suggest that the effect of soft policies have been inflated, the second and the third imply that the proxies used to measure pro- environmental actions miss some important psychological dimensions associated with doing them. Future work should investigate which channel is most likely to be at play.

## Supplementary Information

Supplementary Information.

## Data Availability

The raw data and materials used to conduct this experiment was made publicly available at https://osf.io/2hq84/?view_only=c6f574a40c5248b299081d3ec0d3e411.

## Code availability

The code used to conduct power analyses and the main analyses was made available on at https://osf.io/2hq84/?view_only=c6f574a40c5248b299081d3ec0d3e411.
