# Supplementary material for: Experimental evidence on the role of framing, difficulty and domain-similarity in shaping behavioral spillovers
Source: Sci Rep. 2024 Oct 24;14:25124. doi: 10.1038/s41598-024-71988-x (PMC11502804; doi:10.1038/s41598-024-71988-x)
Supplement: Supplementary file 1 — Supplementary Information. [file 41598_2024_71988_MOESM1_ESM.docx]

**Supplementary information:** Experimental evidence on the role of framing, difficulty and domain-similarity in shaping behavioral spillovers

Anja Köbrich León^1^, Julien Picard^2*^, Janosch Schobin^1^

^1^ University of Kassel, Kassel, Germany

^2^ London School of Economics and Political Science, London, UK

* Corresponding author: Julien Picard ([j.r.picard@lse.ac.uk)](mailto:j.r.picard@lse.ac.uk))

***Appendix A. Experimental setup***

***Experimental setup: Pre-treatment questionnaire online survey (translated in English)***

***Informed Consent***

Welcome and thank you for taking part in our survey. The survey is part of a research project at the University of Kassel. The survey is being conducted by Julien Picard (London School of Economics), Dr. Anja Köbrich Leon (University of Kassel) and Dr. Janosch Schobin (University of Kassel). The objective is to learn more about various current topics concerning the environment.

The survey will take no more than 15 minutes to complete. First, we will begin by asking you some questions about yourself. In particular, about your socio-demographic profile, interests and preferences. Following this, we will invite you to read a short text related to today's eating habits. We will proceed by inviting you to help us with the evaluation of food images. Finally, some participants have the opportunity to address a message to the political representatives of Germany regarding potential environmental policy measures.

Your participation in this survey is completely voluntary and you may opt out of any question in the survey. All of your responses will be kept confidential. They will only be used for statistical purposes and will be reported only in aggregated form. An Institutional Review Board responsible for human subjects research reviewed this research project and found it to be acceptable, according to applicable state and federal regulations designed to protect the rights and welfare of participants in research. We guarantee that the statutory data protection regulations will be strictly observed. Furthermore, we assure you that neither your name nor the associated data will be passed on to third parties.

Please note that you will not be able to return to a previous question during the survey. Therefore, we ask you to read the questions carefully before answering.

If you have any questions about this survey, or difficulty in accessing the site or completing the survey, please contact Anja Köbrich Leon (

[anja.koebrich@uni-kassel.de)](mailto:anja.koebrich@uni-kassel.de))

Thank you in advance for your participation - your opinion is very important to us!

Your University of Kassel

To start the survey, please accept our privacy policy.

**Show privacy policy**

**Privacy policy**

**Data protection information according to Art. 13 DSGVO**

on the occasion of data collection during the participation and implementation of the research project "GREEN" of the department "Empirical Economic Research" of the University of Kassel.

**Responsible in the sense of data protection law:**

University of Kassel

The President

34109 Kassel

Phone: +49-561-804-0

Web: www.uni-kassel.de

E-mail: sekretariat-praesidentin@uni-kassel.de

**Contact details of the data protection officer:**

The data protection officer

University of Kassel

34109 Kassel

Phone: +49-561-804-7036

Web: www.uni-kassel.de/go/datenschutz

E-mail: datenschutz@uni-kassel.de

**Contact details of the responsible institute and contact person:**

Dr. Anja Köbrich Leon

University of Kassel

Department of Empirical Economic Research

Nora-Platiel-Str. 4

34109 Kassel

Phone: +49 561 804-7919

Web: www.uni-kassel.de/fb07/ivwl/empirische-wirtschaftsforschung

E-mail: anja.koebrich@uni-kassel.de

**Purposes of processing**

Your personal data will be processed exclusively for research purposes within the framework of the "GREEN" project. For information on this, please refer to the description of the research project at the following link:

https://www.uni-kassel.de/fb07/ivwl/empirische-wirtschaftsforschung/forschung/forschungsprojekte

**Legal basis for the processing**

The legal basis for the processing of your personal data is your consent pursuant to Art. 6 para. 1 UAbs.1 a) DSGVO. If special categories of personal data are involved, the data we collect will be processed on the basis of your explicit consent pursuant to Art. 9 (2) a) DSGVO.

**Categories of personal data processed:**

- IP address when conducting the survey
- Socio-demographic characteristics (e.g. age, gender, educational attainment, place of residence (zip code), income).
- Attitudes towards and behavior in the area of environmental and climate protection (e.g. environmental awareness)
- Last name, first name and address in the list of co-signers, that will be sent to the German Bundestag

**Recipients or categories of recipients/third party transfer**

Your personal data is or may be transferred to the following recipients: A service provider is used for hosting the system; there is a corresponding contract for order processing. There is no transmission to third countries.

When deciding to join a petition, a behavior under investigation in this survey, we record your last name, first name and address in the list of co-signers, that will be sent to the German Bundestag.

**Duration of storage of personal data/criteria for determining the duration**

- IP addresses - immediate deletion after completion of the survey
- Last name, first name and address in the list of co-signers, that will be sent to the German Bundestag - immediate deletion after sending the list to the German Bundestag
- Socio-demographic characteristics and attitudes towards and behavior in the area of environmental and climate protection - no further deletion on the part of the data controller takes place in accordance with Article 17(3)(d) DSGVO. A review of the data will take place 10 years after the end of the project.

**No automated decision-making (including profiling).**

Processing of your personal data for the purpose of automated decision-making (including profiling) pursuant to Article 22 (1) DSGVO does not take place.

**Right of withdrawal in case of consent**

You have the right to revoke your consent for the future at any time vis-à-vis the named contact person, unless the data has already been anonymized. The legality of the data processing carried out on the basis of the consent until the revocation is not affected by this.

**Your further rights**

You can exercise the following rights at any time vis-à-vis the named contact person or our data protection officers:

- Information about your data stored by us and its processing.
- Correction of incorrect personal data
- Deletion of the personal data concerning you, provided that the conditions for deletion are met
- Restriction of data processing, if we are not yet allowed to delete your data due to legal obligations
- Objection to the processing of your data by us

Please contact us at the following e-mail address: anja.koebrich@uni-kassel.de.

**Right of complaint**

Finally, you have the right to complain to the competent supervisory authority in the event of data protection-related problems.

Contact address of the supervisory authority of the University of Kassel:

The Hessian Commissioner for Data Protection and Freedom of Information (HBDI).

P.O. Box 3163

65021 Wiesbaden

E-mail: poststelle@datenschutz.hessen.de

Telephone: +49-611-1408-0

*Attention checks*

People are very busy these days and many do not have time to follow what goes on in the government. We are testing whether people read questions. To show that you’ve read this much, answer “very interested”:

- Not at all interested
- Slightly interested
- Moderately interested
- Very interested
- Extremely interested

|  |
| --- |

Most modern theories of decision making recognize that decisions do not take place in a vacuum. Individual preferences and knowledge, along with situational variables can greatly impact the decision process. To demonstrate that you’ve read this much, just go ahead and select red among the alternatives below.

- Blue
- Red
- Green
- Yellow
- White

*Demographics*

*Age*

How old are you?

- Under 18
- 18-24 years old
- 25-34 years old
- 35-44 years old
- 45-54 years old
- 55-64 years old
- 65+ years old

*Gender*

*How do you describe yourself?*

- Male
- Female
- Non-binary / third gender
- Prefer to self-describe
- Prefer not to say

*Location*

Please indicate the zip code of your primary residence:

_____ Zip Code

*Education*

What is the highest school or college degree you have completed?

- I left school without a diploma
- I am currently going to school
- I am currently studying
- Volks- / Hauptschulabschluss (GDR: 8th grade)
- Realschulabschluss / Mittlere Reife (GDR: 10th grade)
- Fachhochschulreife (completion of a specialized secondary school)
- Abitur/university entrance qualification
- Fachhochschule or Berufsakademie degree (GDR: engineering and technical college degree)
- University or college degree
- Doctorate or habitation
- Other degree, namely:

*Income*

Information about income is very important to understand.  Would you please give your best guess? Please indicate the answer that includes your entire household income in (previous year) before taxes.

- Less than €10,000
- €10,000 to €19,999
- €20,000 to €29,999
- €30,000 to €39,999
- €40,000 to €49,999
- €50,000 to €59,999
- €60,000 to €69,999
- €70,000 to €79,999
- €80,000 to €89,999
- €90,000 to €99,999
- €100,000 to €149,999
- €150,000 or more

*Pro-Environmental and altruistic values*

In what follows, you will be shown the values hold by someone. Please indicate the extent to which you share these values. There is not right or wrong answer.

| Values *(shown in randomized order)* | Very much like me | Like me | Somewhat like me | Neither like me nor not like me | Somewhat not like me | Not like me | Very much not like me |
| --- | --- | --- | --- | --- | --- | --- | --- |
| It is important for him/her to prevent environmental pollution. | □ | □ | □ | □ | □ | □ | □ |
| It is important for him/her to protect the environment. | □ | □ | □ | □ | □ | □ | □ |
| It is important for him/her to respect nature. | □ | □ | □ | □ | □ | □ | □ |
| It is important for him/her to be in harmony with nature. | □ | □ | □ | □ | □ | □ | □ |
| It is important for him/her that every person has the same opportunities. | □ | □ | □ | □ | □ | □ | □ |
| It is important for him/her to care about those who are worse off than him/her. | □ | □ | □ | □ | □ | □ | □ |
| It is important to him/her that everyone is treated fairly. | □ | □ | □ | □ | □ | □ | □ |
| It is important for him/her that there is no war or conflict. | □ | □ | □ | □ | □ | □ | □ |
| It is important for him/her to be helpful to others. | □ | □ | □ | □ | □ | □ | □ |

***Guilt proneness***

Please describe the extent to which you agree with the following statements. There is no right or wrong answer.

| Statement *(shown in randomized order)* | Very unlikely | Unlikely | Slightly unlikely | Not likely nor unlikely | Slightly likely | Likely | Very likely |
| --- | --- | --- | --- | --- | --- | --- | --- |
| After realizing you have received too much change at a store, you decide to keep it because the salesclerk doesn’t notice. What is the likelihood that you would feel uncomfortable about keeping the money? | □ | □ | □ | □ | □ | □ | □ |
| You are privately informed that you are the only one in your group that skipped too many days of school. What is the likelihood that this would lead you to become more responsible about attending school? | □ | □ | □ | □ | □ | □ | □ |
| You reveal a friend’s secret, though your friend never finds out. What is the likelihood that your failure to keep the secret would lead you to exert extra effort to keep secrets in the future? | □ | □ | □ | □ | □ | □ | □ |
| You strongly defend a point of view in a discussion, and though nobody was aware of it, you realize that you were wrong. What is the likelihood that this would make you think more carefully before you speak? | □ | □ | □ | □ | □ | □ | □ |
| At a coworker’s housewarming party, you spill red wine on their new cream-colored carpet. You cover the stain with a chair so that nobody notices your mess. What is the likelihood that you would feel that the way you acted was pathetic? | □ | □ | □ | □ | □ | □ | □ |
| While discussing a heated subject with friends, you suddenly realize you are shouting though nobody seems to notice. What is the likelihood that you would try to act more considerately toward your friends? | □ | □ | □ | □ | □ | □ | □ |

*Political spectrum*

In political matters, people talk of "the left" and "the right." How would you place your views generally speaking?

|  | Left | Centre | Right | Skip question |
| --- | --- | --- | --- | --- |
|  | 0 | 5 | 10 |  |

|  |  |  |
| --- | --- | --- |

|  | 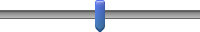 |
| --- | --- |

***Experimental setup: Treatment texts – Study 1 (translated into English)***

***Group 1: text displayed to the placebo group***


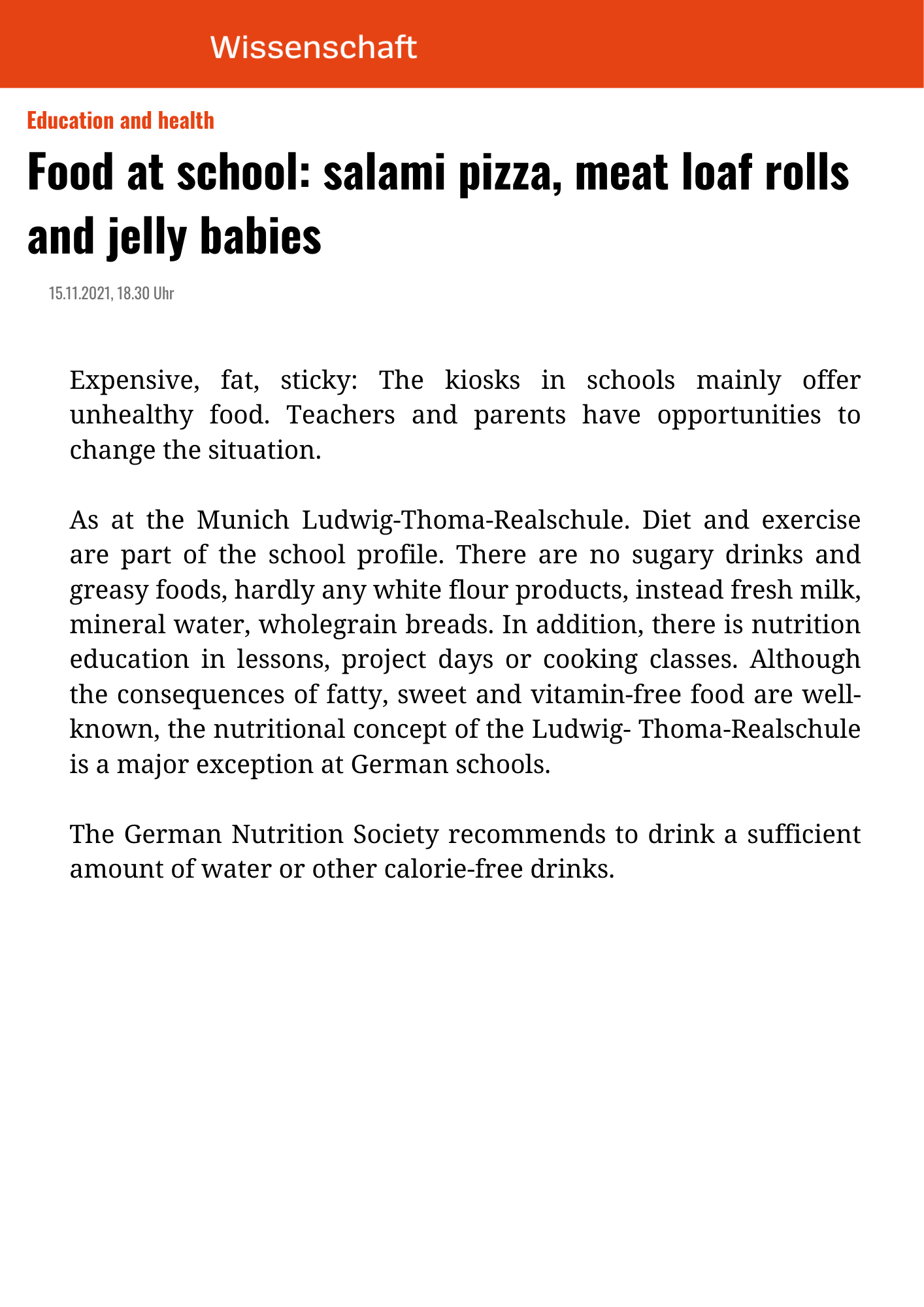


***Group 2: text displayed to the control group***


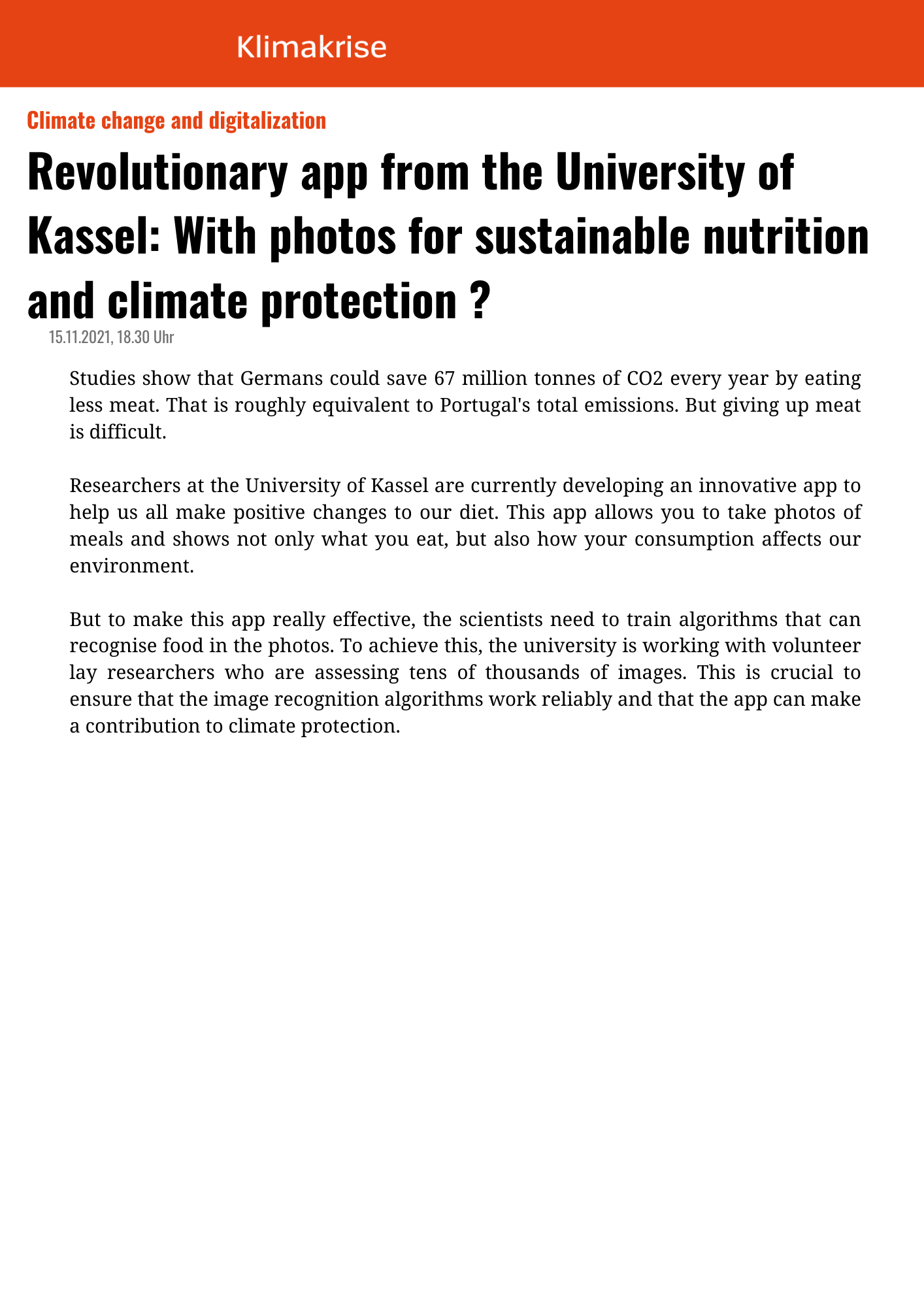


***Group 3: text displayed to the group allocated to the “doom-and-gloom” narrative***


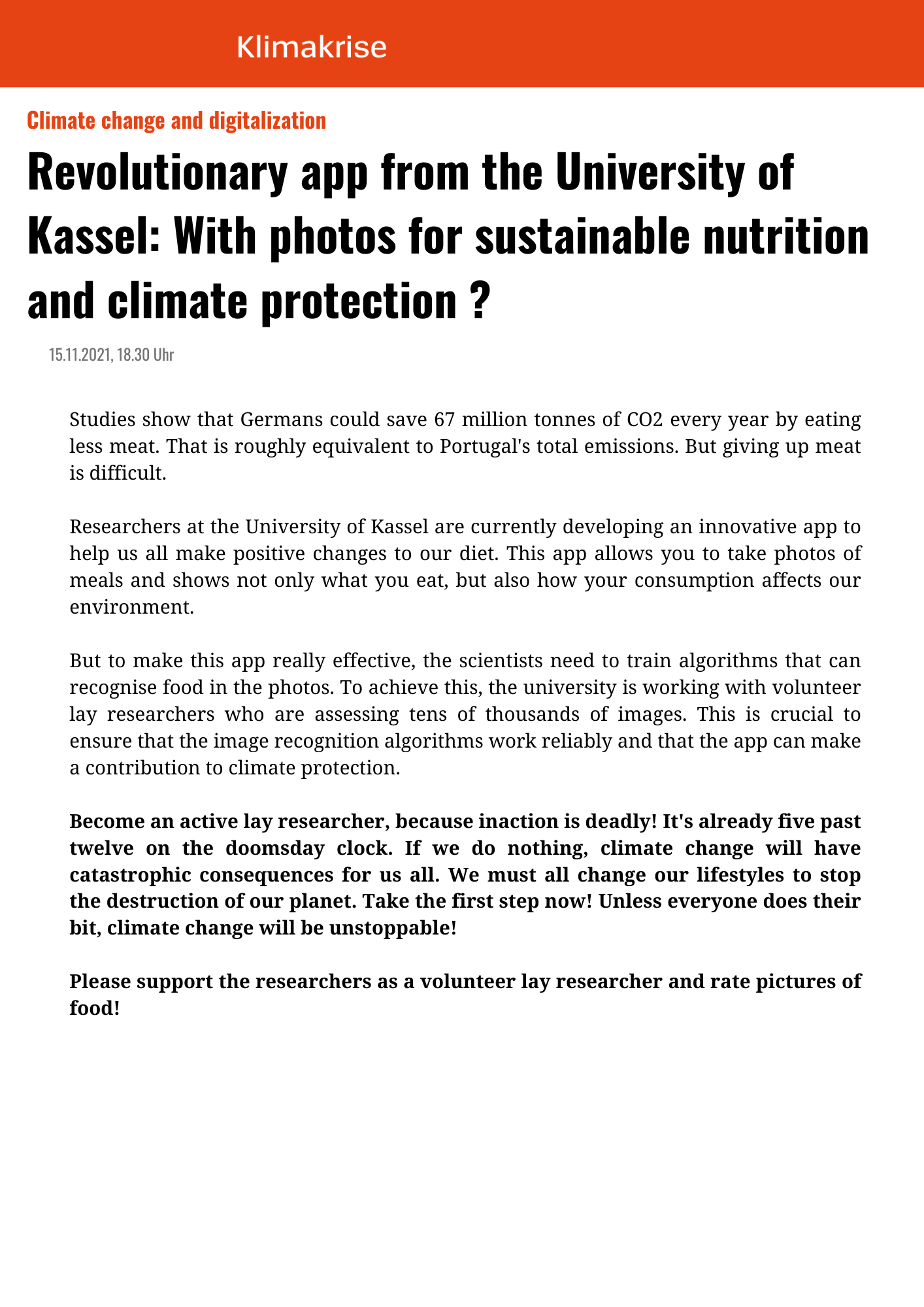


***Group 4: text displayed to the group allocated to the “win-win” narrative***


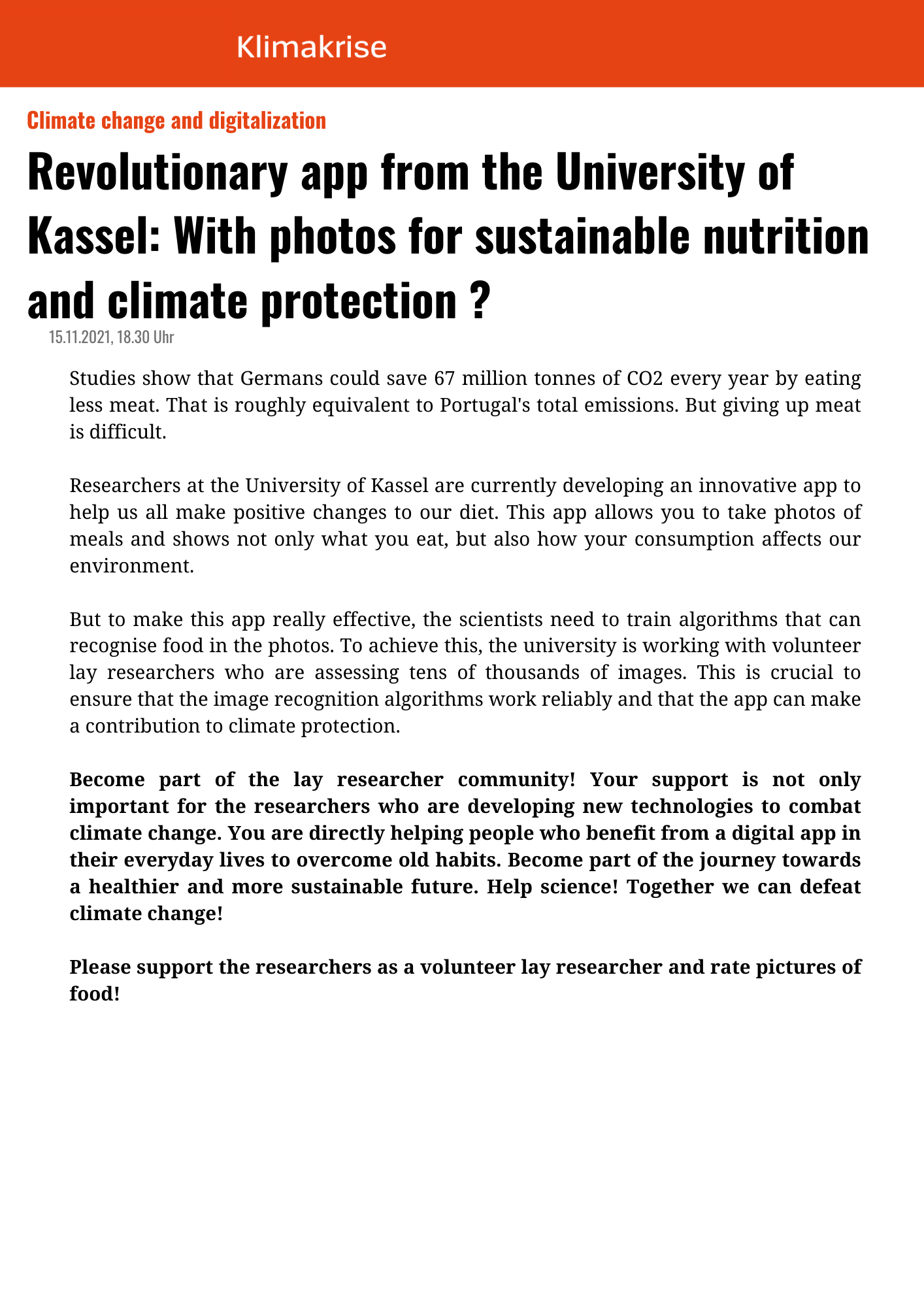


***Experimental setup: PEB1 (translated into English)***

***C.1: Instrumental variable: choice architecture default nudge***

Facilitating choice architecture


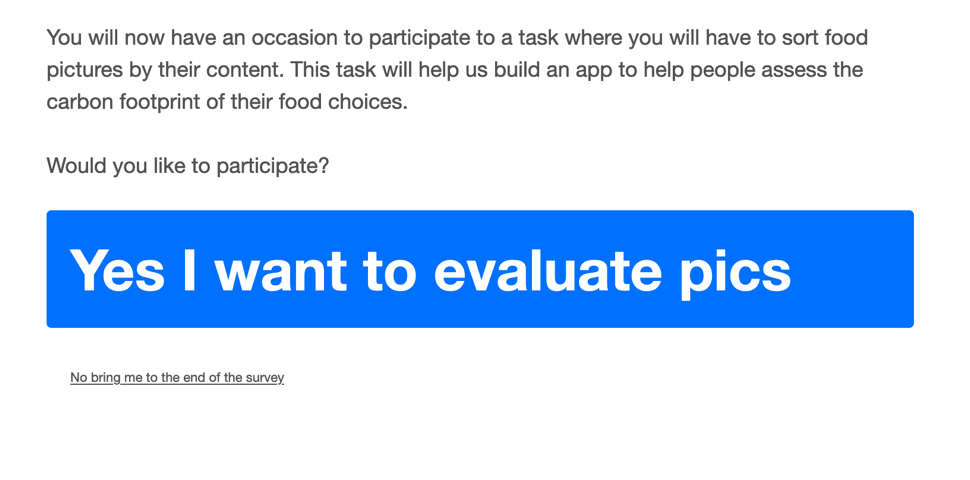


Hardening choice architecture


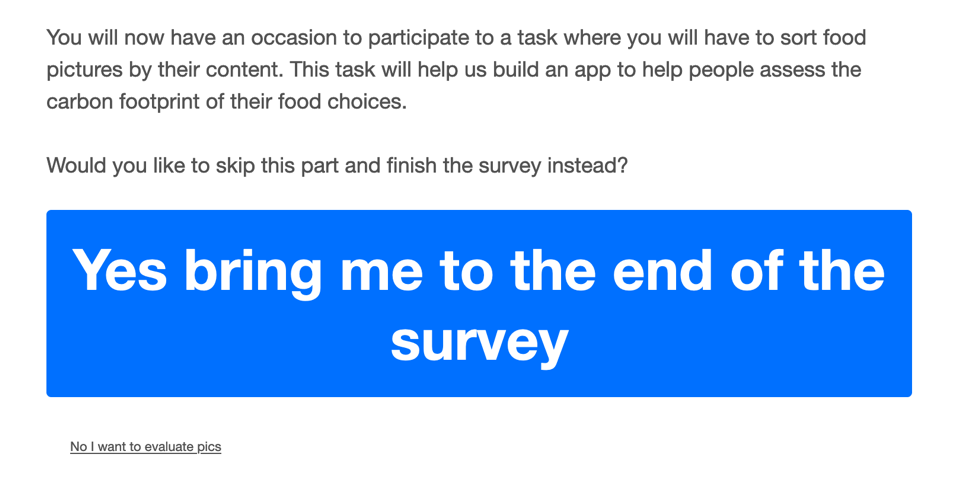


***C.2: Instructions for PEB1***


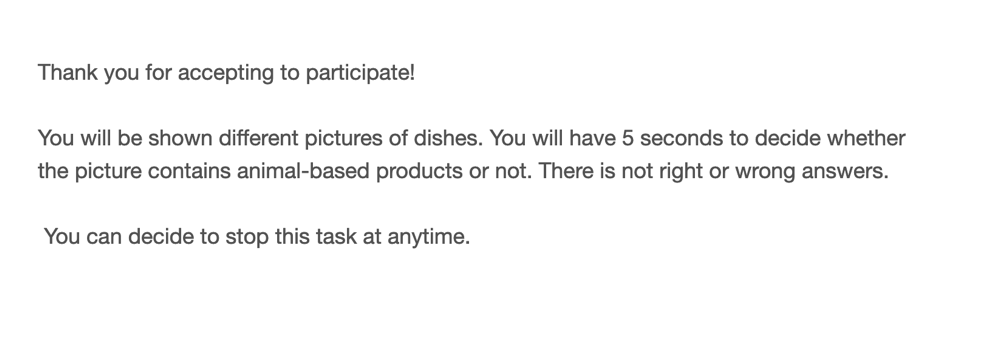


***C.3 PEB1***

Easy PEB1 with hardening choice architecture


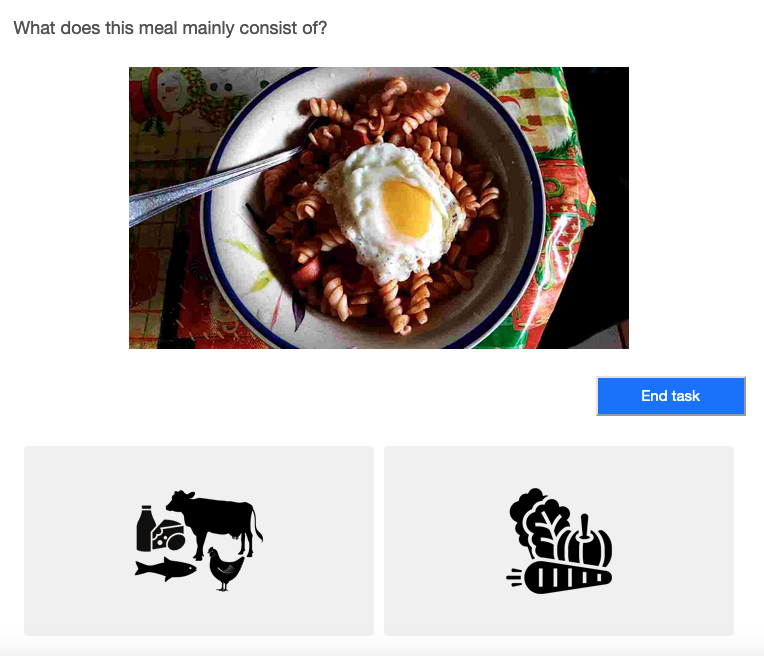


Easy PEB1 with facilitating choice architecture


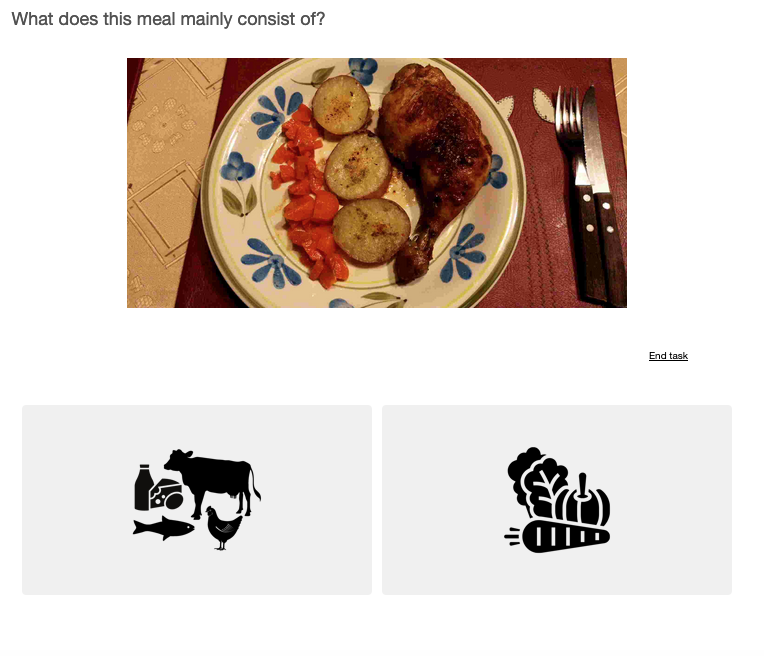


Hard PEB1 with hardening choice architecture


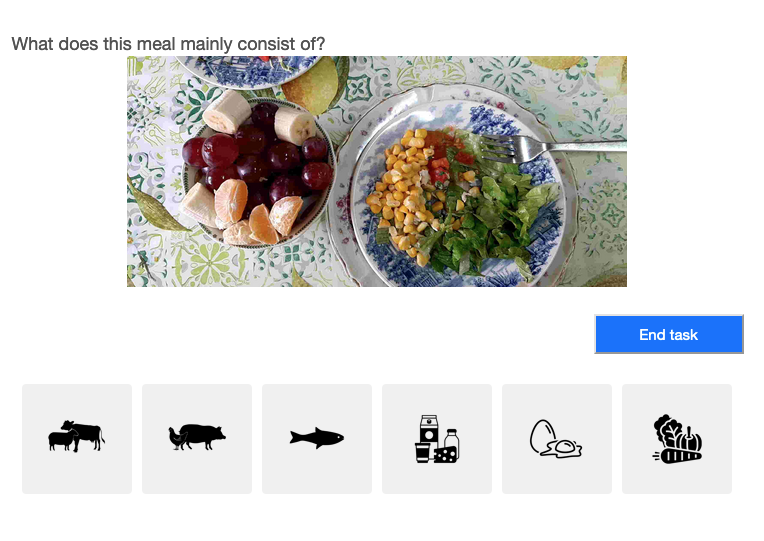


Hard PEB1 with facilitating choice architecture


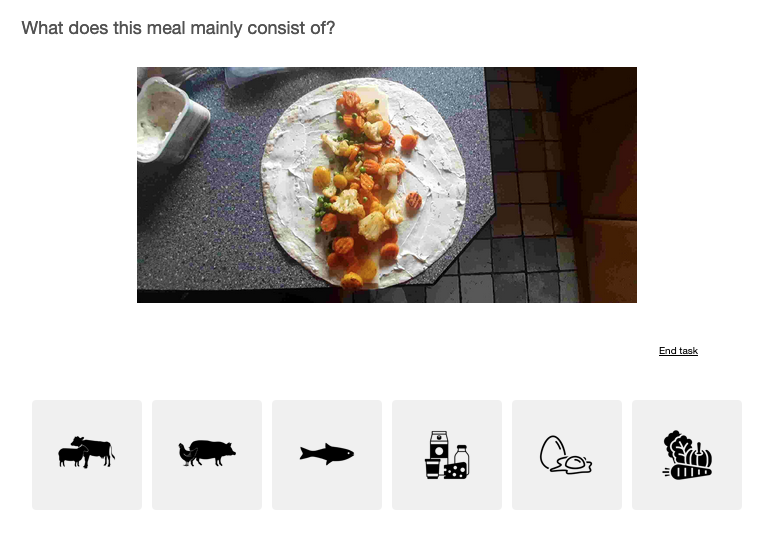


***Experimental setup: Filler tasks***

Move the sliders below to match the percentage displayed on the left. For instance, 0% means that the slider is at the left tip of the scale while 100% means that the slider is at the right tip of the scale.

|  | 0 | 100 |
| --- | --- | --- |

| 9% | 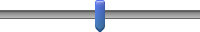 |
| --- | --- |
| 45% | 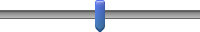 |
| 50% | 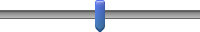 |
| 86% | 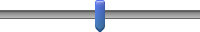 |
| 90% | 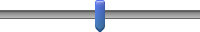 |

Move the pictures to the category you think is the most appropriate.

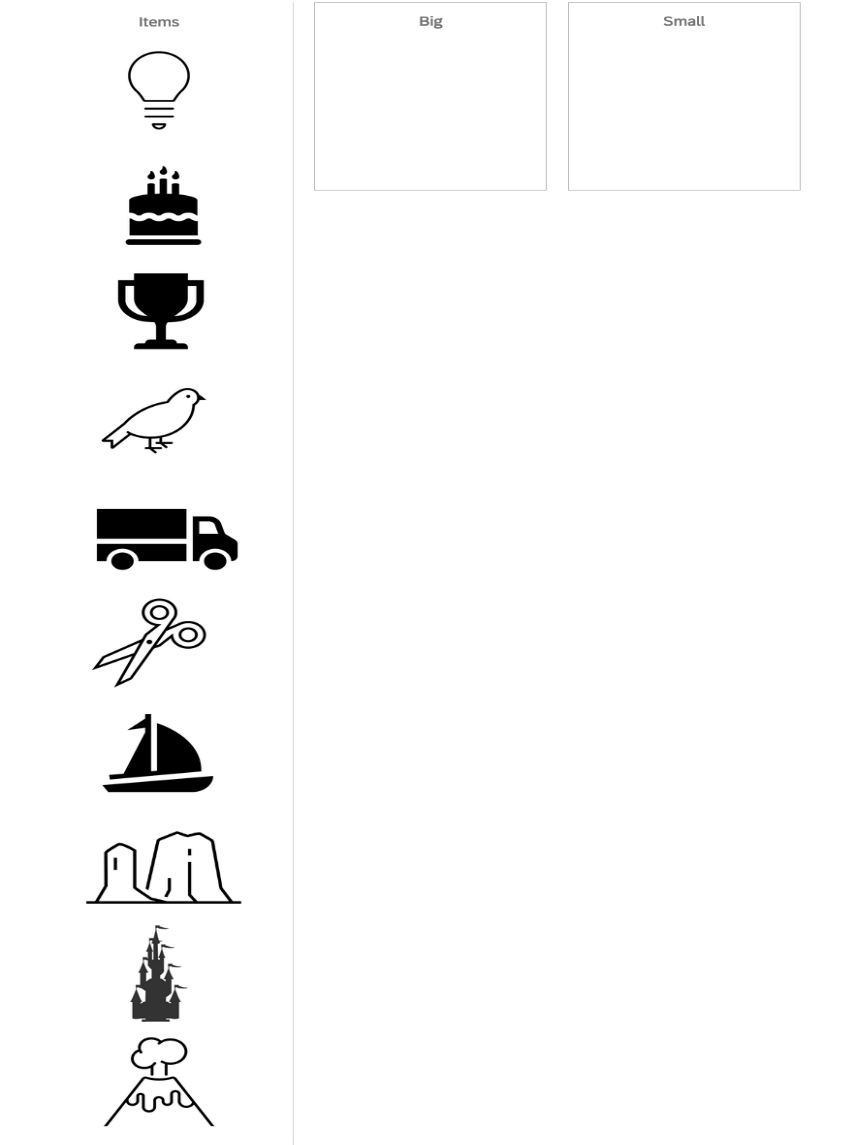


***Experimental setup: PEB2 (translated into English)***

***PEB2a: Petition 1 - same-domain PEB2***

Private road transport

Linking subsidies for the purchase of low-emission vehicles to CO_2_ emissions

Text of the petition

The petition calls for subsidies for the purchase of low-emission vehicles to be linked to the CO_2_ emissions of high-emission vehicles.

Rationale

To increase demand for electric vehicles and high-efficiency vehicles in Germany, CO_2_-based financial incentives for vehicle purchases should be introduced. Buyers of a vehicle with low CO_2_ emissions will continue to receive a subsidy, which, however, will be covered by those buying a particularly CO_2_-intensive vehicle.

***PEB2b: Petition 2 - different-domain PEB2***

Personal preventive health care

Nationwide campaign to address social isolation and loneliness among adolescents

Text of the petition

The petition calls for a nationwide campaign to be launched to offer concrete recommendations and assistance to young people experiencing feelings of loneliness and social isolation.

Rationale

Loneliness and social isolation is not just a challenge that affects older people in Germany. In particular, the age group between 18 and 29 is very strongly affected. There are virtually no nationwide efforts that address this issue.

Therefore, it is appropriate to expand existing efforts to make the issue visible to the age group of adolescents as well. Although doing so will not eradicate the threat, it will certainly be effective in mitigating it, and on a sustained basis. Scientific studies have shown that the earlier loneliness is experienced, the greater the likelihood that it will become a permanent condition.

***Experimental setup: Study objective information at the end (translated into English)***

Thank you for your participation.

The purpose of our study was to explore the extent to which information on the role of science in combating climate change encourages you to contribute by supporting science in evaluating food images. Food choices are an important lever to mitigate climate change.

In addition, we wanted to explore whether you would be willing to take an active role in helping the environment. We were also interested in knowing how the phrasing of information on the role of science to fight climate change affected your decision.

Your answers are fully anonymized and will only be used for research purposes. However, if you would like to withdraw from this study, please click on the ‘withdraw’ button below. Otherwise, click on the ‘next’ button.

***Appendix B. Robustness check***

Table B.1: Robustness checks - Hypothesis 1

| Data included | Study 1 | Study 1 | Study 1 | Study 1+2+3 | Study 1 | Study 1 | Study 1+2+3 |
| --- | --- | --- | --- | --- | --- | --- | --- |
| Outcome | PEB1 (binary) | | | | PEB1 (continuous) | | |
| Model | Probit | OLS | OLS | OLS | OLS | OLS | OLS |
|  | (1) | (2) | (3) | (4) | (5) | (6) | (7) |
| Constant |  | 0.553*** | 0.197** | 0.465*** | 15.195*** | 4.582 | 12.300*** |
|  |  | (0.051) | (0.096) | (0.032) | (1.516) | (2.837) | (0.943) |
| Salience nudge | 0.340*** | 0.356*** | 0.359*** | 0.365*** | 10.692*** | 10.796*** | 11.003*** |
|  | (0.013) | (0.017) | (0.017) | (0.012) | (0.512) | (0.508) | (0.344) |
| Win-Win vs. Control | -0.025 | -0.026 | -0.024 | -0.020 | -0.648 | -0.600 | -0.354 |
|  | (0.016) | (0.017) | (0.017) | (0.016) | (0.512) | (0.509) | (0.477) |
| Control for social-demographics |  | X | X | X | X | X | X |
| Control for attitudes |  |  | X |  |  | X |  |
| Number of observations | 2727 | 2356 | 2356 | 5228 | 2356 | 2356 | 5228 |
| R2 |  | 0.171 | 0.180 | 0.165 | 0.175 | 0.185 | 0.170 |
| *Note: This table presents different specifications estimating the ATE of win-win arguments on participation in PEB1. When using the full sample, we add studies fixed-effects. Robust standard errors are in parentheses. * p < 0.1, ** p < 0.05, *** p < 0.01.* p < 0.1, ** p < 0.05, *** p < 0.01.* | | | | | | | |

Table B.2: Robustness checks - Hypothesis 2

| Data included | Study 1 | Study 1 | Study 1 | Study 1+2+3 | Study 1 | Study 1 | Study 1+2+3 |
| --- | --- | --- | --- | --- | --- | --- | --- |
| Outcome | PEB1 (binary) | | | | PEB1 (continuous) | | |
| Model | Probit | OLS | OLS | OLS | OLS | OLS | OLS |
|  | (1) | (2) | (3) | (4) | (5) | (6) | (7) |
| Constant |  | 0.518*** | 0.278*** | 0.438*** | 14.036*** | 5.750** | 11.348*** |
|  |  | (0.051) | (0.099) | (0.026) | (1.512) | (2.873) | (0.781) |
| Salience nudge | 0.355*** | 0.376*** | 0.376*** | 0.371*** | 11.489*** | 11.487*** | 11.250*** |
|  | (0.012) | (0.017) | (0.017) | (0.009) | (0.505) | (0.503) | (0.282) |
| Doom & Gloom vs. Control | -0.025 | -0.023 | -0.024 | -0.025*** | -0.410 | -0.466 | -0.613** |
|  | (0.016) | (0.017) | (0.017) | (0.009) | (0.511) | (0.509) | (0.282) |
| Control for social-demographics |  | X | X | X | X | X | X |
| Control for attitudes |  |  | X |  |  | X |  |
| Number of observations | 2760 | 2358 | 2358 | 7843 | 2358 | 2358 | 7843 |
| R2 |  | 0.182 | 0.186 | 0.168 | 0.190 | 0.197 | 0.174 |
| *Note: This table presents different specifications estimating the ATE of doom-and-gloom arguments on participation in PEB1. When using the full sample, we add studies fixed-effects. Robust standard errors are in parentheses. * p < 0.1, ** p < 0.05, *** p < 0.01.* p < 0.1, ** p < 0.05, *** p < 0.01.* | | | | | | | |
|  |  |  |  |  |  |  |  |

Table B.3: Robustness checks - Hypothesis 3

| Data included | Study 1 | Study 1 | Study 1 | Study 1+2+3 | Study 1 | Study 1 | Study 1 | Study 1+2+3 |
| --- | --- | --- | --- | --- | --- | --- | --- | --- |
| Outcome | PEB2 | | | | | | | |
| PEB1 | Binary | | | | Continuous | | | |
| Model | R&V (1998) | OLS | OLS | OLS | R&V (1998) | OLS | OLS | OLS |
|  | (1) | (2) | (3) | (4) | (5) | (6) | (7) | (8) |
| Constant |  | 0.255*** | -0.307*** | 0.168*** |  | 0.254*** | -0.308*** | 0.167*** |
|  |  | (0.045) | (0.070) | (0.028) |  | (0.045) | (0.070) | (0.027) |
| PEB1 | -0.010 | -0.023 | -0.017 | -0.008 | 0.000 | -0.001 | -0.001 | 0.000 |
|  | (0.031) | (0.034) | (0.033) | (0.026) | (0.001) | (0.001) | (0.001) | (0.001) |
| All vs. Placebo | 0.025* | 0.025* | 0.021 | 0.025* | 0.025* | 0.025* | 0.021 | 0.025* |
|  | (0.014) | (0.015) | (0.014) | (0.013) | (0.014) | (0.015) | (0.014) | (0.013) |
| Control for social-demographics |  | X | X | X |  | X | X | X |
| Control for attitudes |  |  | X |  |  |  | X |  |
| Number of observations | 5492 | 4704 | 4704 | 8112 | 5492 | 4704 | 4704 | 8112 |
| *Note: This table presents different specifications estimating the effect of participating in the real-effort task (instrumented by allocation to the salience nudge) on respondents' likelihood of signing the environment-related petition, controlling for their allocation to one of the treatment texts. When using the full sample, we add studies fixed-effects. Robust standard errors are in parentheses. * p < 0.1, ** p < 0.05, *** p < 0.01.* p < 0.1, ** p < 0.05, *** p < 0.01.* | | | | | | | | |

Table B.4: Robustness checks - Hypothesis 4

|  | Study 1 | Study 1 | Study 1 | Study 1+2+3 | Study 1 | Study 1 | Study 1 | Study 1+2+3 |
| --- | --- | --- | --- | --- | --- | --- | --- | --- |
| Outcome | PEB2 | | | | | | | |
| PEB1 | Binary | | | | Continuous | | | |
| Model | Probit | OLS | OLS | OLS | Probit | OLS | OLS | OLS |
|  | (1) | (2) | (3) | (4) | (5) | (6) | (7) | (8) |
| Constant |  | 0.354*** | -0.170* | 0.206*** |  | 0.352*** | -0.172* | 0.204*** |
|  |  | (0.067) | (0.097) | (0.041) |  | (0.066) | (0.097) | (0.039) |
| PEB1 | -0.041 | -0.062 | -0.043 | -0.022 | -0.001 | -0.002 | -0.001 | -0.001 |
|  | (0.046) | (0.052) | (0.050) | (0.038) | (0.002) | (0.002) | (0.002) | (0.001) |
| Win-Win vs. Control | 0.003 | 0.004 | 0.004 | 0.002 | 0.003 | 0.004 | 0.004 | 0.002 |
|  | (0.017) | (0.019) | (0.018) | (0.017) | (0.017) | (0.019) | (0.018) | (0.017) |
| Control for social-demographics |  | X | X | X |  | X | X | X |
| Control for attitudes |  |  | X |  |  |  | X |  |
| Number of observations. | 2727 | 2356 | 2356 | 4022 | 2727 | 2356 | 2356 | 4022 |
| *Note: This table presents different specifications estimating the direct effect of win-win arguments on respondents' likelihood of signing the environment-related petition, controlling for participation in the real effort task (instrumented by allocation to the salience nudge). When using the full sample, we add studies fixed-effects. Robust standard errors are in parentheses. * p < 0.1, ** p < 0.05, *** p < 0.01. * p < 0.1, ** p < 0.05, *** p < 0.01.* | | | | | | | | |

Table B.5: Robustness checks - Hypothesis 5

| Data included | Study 1 | Study 1 | Study 1 | Study 1+2+3 | Study 1 | Study 1 | Study 1 | Study 1+2+3 |
| --- | --- | --- | --- | --- | --- | --- | --- | --- |
| Outcome | PEB2 | | | | | | | |
| PEB1 | Binary | | | | Continuous | | | |
| Model | Probit | OLS | OLS | OLS | Probit | OLS | OLS | OLS |
|  | (1) | (2) | (3) | (4) | (5) | (6) | (7) | (8) |
| Constant |  | 0.290*** | -0.403*** | 0.186*** |  | 0.287*** | -0.408*** | 0.184*** |
|  |  | (0.065) | (0.101) | (0.034) |  | (0.063) | (0.100) | (0.032) |
| PEB1 | -0.037 | -0.051 | -0.052 | -0.021 | -0.001 | -0.002 | -0.002 | -0.001 |
|  | (0.044) | (0.049) | (0.047) | (0.033) | (0.001) | (0.002) | (0.002) | (0.001) |
| Doom & Gloom vs. Control | -0.011 | -0.007 | -0.011 | -0.001 | -0.010 | -0.006 | -0.011 | -0.001 |
|  | (0.017) | (0.019) | (0.018) | (0.012) | (0.017) | (0.018) | (0.018) | (0.012) |
| Control for social-demographics |  | X | X | X |  | X | X | X |
| Control for attitudes |  |  | X |  |  |  | X |  |
| Number of observations | 2760 | 2358 | 2358 | 5380 | 2760 | 2358 | 2358 | 5380 |
| *Note: This table presents different specifications estimating the direct effect of doom-and-gloom arguments on respondents' likelihood of signing the environment-related petition, controlling for participation in the real-effort task (instrumented by allocation to the salience nudge). When using the full sample, we add waves fixed-effects. Robust standard errors are in parentheses. * p < 0.1, ** p < 0.05, *** p < 0.01.* p < 0.1, ** p < 0.05, *** p < 0.01.* | | | | | | | | |

Table B.6: Robustness checks - Hypothesis 6

| Data included | Study 1+2 | | | | | |
| --- | --- | --- | --- | --- | --- | --- |
| Outcome | PEB2 | | | | | |
| PEB1 | Binary | | | Continuous | | |
| Model | Probit | OLS | OLS | Probit | OLS | OLS |
|  | (1) | (2) | (3) | (4) | (5) | (6) |
| Constant |  | 0.244*** | -0.310*** |  | 0.243*** | -0.311*** |
|  |  | (0.040) | (0.062) |  | (0.039) | (0.061) |
| PEB1 | -0.012 | -0.023 | -0.019 | 0.000 | -0.001 | -0.001 |
|  | (0.032) | (0.034) | (0.033) | (0.001) | (0.001) | (0.001) |
| Difficulty | -0.002 | 0.015 | 0.013 | -0.001 | 0.016 | 0.013 |
|  | (0.043) | (0.048) | (0.047) | (0.041) | (0.046) | (0.045) |
| All vs. Placebo | 0.025* | 0.025* | 0.020 | 0.025* | 0.025* | 0.021 |
|  | (0.014) | (0.015) | (0.014) | (0.014) | (0.015) | (0.014) |
| PEB1 x Difficulty | 0.005 | -0.018 | -0.021 | 0.000 | -0.001 | -0.001 |
|  | (0.059) | (0.065) | (0.064) | (0.002) | (0.002) | (0.002) |
| Control for social-demographics |  | X | X |  | X | X |
| Control for attitudes |  |  | X |  |  | X |
| Number of observations | 8112 | 6874 | 6855 | 8112 | 6874 | 6855 |
| *Note: This table presents different specifications estimating the effect of varying the difficulty of the real-effort task on the effect of doing the real-effort task (instrumented by allocation to the salience nudge) on the likelihood of signing the environment-related petition. * p < 0.1, ** p < 0.05, *** p < 0.01.* p < 0.1, ** p < 0.05, *** p < 0.01.* | | | | | | |

Table B.7: Robustness checks - Hypothesis 7 and Hypothesis 8

| Data included | Study 1+2 | | | | | |
| --- | --- | --- | --- | --- | --- | --- |
| Outcome | PEB2 | | | | | |
| PEB1 | Binary | | | Continuous | | |
| Model | Probit | OLS | OLS | Probit | OLS | OLS |
|  | (1) | (2) | (3) | (4) | (5) | (6) |
| Constant |  | 0.317*** | -0.317*** |  | 0.314*** | -0.321*** |
|  |  | (0.042) | (0.065) |  | (0.041) | (0.064) |
| PEB1 | -0.025 | -0.045 | -0.045 | 0.001 | -0.001 | -0.001 |
|  | (0.034) | (0.037) | (0.036) | (0.001) | (0.001) | (0.001) |
| Petition | 0.016 | -0.016 | -0.025 | 0.057 | -0.011 | -0.019 |
|  | (0.045) | (0.053) | (0.052) | (0.036) | (0.051) | (0.050) |
| Doom & Gloom vs. Control | -0.001 | -0.003 | -0.002 | 0.000 | -0.003 | -0.002 |
|  | (0.013) | (0.013) | (0.013) | (0.013) | (0.013) | (0.013) |
| PEB1 x Petition | 0.101* | 0.138** | 0.156** | 0.002 | 0.005** | 0.005** |
|  | (0.058) | (0.070) | (0.069) | (0.002) | (0.002) | (0.002) |
| Doom & Gloom vs. Control x Petition | 0.038* | 0.054** | 0.057** | 0.036* | 0.054** | 0.057** |
|  | (0.022) | (0.025) | (0.025) | (0.022) | (0.025) | (0.025) |
| Control for social-demographics |  | X | X |  | X | X |
| Control for attitudes |  |  | X |  |  | X |
| Number of observations | 7936 | 6628 | 6609 | 7936 | 6628 | 6609 |
| *Note: This table presents different specifications estimating the effect of varying the cause supported by the petition on the effect of doing the real-effort task (instrumented by allocation to the salience nudge) and the effect of doom-and-gloom arguments on the likelihood of signing the petition. For all specifications, we control for the difficulty of PEB1. Robust standard errors are in parentheses. * p < 0.1, ** p < 0.05, *** p < 0.01.* p < 0.1, ** p < 0.05, *** p < 0.01.* | | | | | | |

***Appendix C. Further Exploratory Analyses***

Table C.1: Heterogeneity analysis - Hypothesis 1 and 2

| Data included | Study 1 without placebo and doom-and-gloom groups | | | | Study 1 without placebo and win-win groups | | | |
| --- | --- | --- | --- | --- | --- | --- | --- | --- |
| Outcome | PEB1 (binary) | | PEB1 (continuous | | PEB1 (binary) | | PEB1 (continuous) | |
|  | (1) | (2) | (3) | (4) | (5) | (6) | (7) | (8) |
| Constant | 0.468*** | 0.477*** | 11.823*** | 12.260*** | 0.490*** | 0.478*** | 12.682*** | 12.323*** |
|  | (0.021) | (0.020) | (0.602) | (0.564) | (0.020) | (0.021) | (0.582) | (0.617) |
| Salience nudge | 0.370*** | 0.369*** | 11.185*** | 11.151*** | 0.383*** | 0.383*** | 11.710*** | 11.711*** |
|  | (0.016) | (0.016) | (0.475) | (0.475) | (0.016) | (0.016) | (0.470) | (0.469) |
| Win-Win vs. Control | 0.008 | -0.002 | 0.565 | -0.103 |  |  |  |  |
|  | (0.026) | (0.024) | (0.766) | (0.706) |  |  |  |  |
| Altruism | 0.094*** |  | 3.257*** |  |  |  |  |  |
|  | (0.023) |  | (0.669) |  |  |  |  |  |
| Pro-environment |  | 0.091*** |  | 2.927*** |  |  |  |  |
|  |  | (0.022) |  | (0.654) |  |  |  |  |
| Altruism x Win-Win vs. Control | -0.052 |  | -1.683 |  |  |  |  |  |
|  | (0.033) |  | (0.974) |  |  |  |  |  |
| Pro-environment x Win-Win vs. Control |  | -0.042 |  | -0.679 |  |  |  |  |
|  |  | (0.032) |  | (0.950) |  |  |  |  |
| Doom & Gloom vs. Control |  |  |  |  | -0.010 | -0.012 | -0.105 | -0.281 |
|  |  |  |  |  | (0.024) | (0.026) | (0.705) | (0.774) |
| Guilt (NBE) |  |  |  |  | 0.050** |  | 1.494** |  |
|  |  |  |  |  | (0.022) |  | (0.660) |  |
| Guilt (repair) |  |  |  |  |  | 0.063*** |  | 1.907*** |
|  |  |  |  |  |  | (0.023) |  | (0.678) |
| Guilt (NBE) x Doom & Gloom vs. Control |  |  |  |  | -0.025 |  | -0.477 |  |
|  |  |  |  |  | (0.032) |  | (0.948) |  |
| Guilt (repair) x Doom & Gloom vs. Control |  |  |  |  |  | -0.019 |  | -0.143 |
|  |  |  |  |  |  | (0.033) |  | (0.975) |
| Number of observations | 2727 | 2727 | 2727 | 2727 | 2760 | 2760 | 2760 | 2760 |
| R2 | 0.167 | 0.168 | 0.175 | 0.176 | 0.174 | 0.176 | 0.184 | 0.186 |
| *Note: This table displays the effect of respondents' attitudes regarding guilt, altruism and the environment on the ATEs of win-win and doom-and-gloom arguments on PEB1. We use OLS regressions. Columns one to four present the interaction between allocation to win-win arguments and scoring above the median for altruistic and pro-environmental attitudes. Columns five to eight present the interaction between allocation to doom-and-gloom arguments and scoring above the median for guilt-proneness attitudes. NBE stands for Negative Behavior Evaluation. It captures the extent to which one feels bad about one's behavior. "Guilt (Repair)" captures respondents' tendency to act to temper guilt. Robust standard errors are in parentheses. We apply Benjamini and Hochberg^61^ correction to conventional p-values (p). * p < 0.1, ** p < 0.05, *** p < 0.01.* p < 0.1, ** p < 0.05, *** p < 0.01.* | | | | | | | | |

Table C.2: Heterogeneity analysis - Hypothesis 4 and 5

| Data included | Study 1 without placebo and doom-and-gloom groups | | | |
| --- | --- | --- | --- | --- |
| Outcome | PEB1 (binary) | | PEB1 (continuous | |
|  | (1) | (2) | (3) | (4) |
| Constant | 0.202*** | 0.191*** | 0.264*** | 0.229*** |
|  | (0.032) | (0.031) | (0.033) | (0.032) |
| Salience nudge | -0.001 | -0.001 | -0.001 | -0.001 |
|  | (0.002) | (0.002) | (0.001) | (0.001) |
| Win-Win vs. Control | 0.045* | 0.010 |  |  |
|  | (0.025) | (0.021) |  |  |
| Altruism | 0.148*** |  |  |  |
|  | (0.024) |  |  |  |
| Pro-environment |  | 0.191*** |  |  |
|  |  | (0.024) |  |  |
| Altruism x Win-Win vs. Control | -0.071 |  |  |  |
|  | (0.034) |  |  |  |
| Pro-environment x Win-Win vs. Control |  | -0.017 |  |  |
|  |  | (0.033) |  |  |
| Doom & Gloom vs. Control |  |  | -0.026 | -0.003 |
|  |  |  | (0.024) | (0.025) |
| Guilt (NBE) |  |  | 0.052** |  |
|  |  |  | (0.024) |  |
| Guilt (repair) |  |  |  | 0.101*** |
|  |  |  |  | (0.024) |
| Guilt (NBE) x Doom & Gloom vs. Control |  |  | 0.029 |  |
|  |  |  | (0.034) |  |
| Guilt (repair) x Doom & Gloom vs. Control |  |  |  | -0.012 |
|  |  |  |  | (0.034) |
| Number of observations | 2727 | 2727 | 2760 | 2760 |
| *Note: This table displays the effect of respondents' attitudes regarding guilt, altruism and the environment on the direct effects of win-win and doom-and-gloom arguments on PEB2. We use 2SLS regressions where PEB1 is instrumented by allocation to the salience nudge. Columns one to four present the interaction between allocation to win-win arguments and scoring above the median for altruistic and pro-environmental attitudes. Columns five to eight present the interaction between allocation to doom-and-gloom arguments and scoring above the median for guilt-proneness attitudes. NBE stands for Negative-Behavior Evaluation. It captures the extent to which one feels bad about one's behavior. "Guilt (Repair)" captures respondents' tendency to act to temper guilt. Robust standard errors are in parentheses. We apply Benjamini and Hochberg^61^ correction to conventional p-values (p). * p < 0.1, ** p < 0.05, *** p < 0.01.* p < 0.1, ** p < 0.05, *** p < 0.01.* | | | | |

Table C.3: Time Spent Reading the Articles and Treatment Effects

| Data included | Wave 1 | |
| --- | --- | --- |
| Outcome | PEB1 (binary) | PEB1 (continuous) |
|  | (1) | (2) |
| Constant | 0.683*** | 18.649*** |
|  | (0.014) | (0.415) |
| Time reading | 0.004 | 0.118 |
|  | (0.003) | (0.094) |
| All vs. Placebo | 0.014 | 0.522 |
|  | (0.016) | (0.473) |
| Time reading x All vs. Placebo | -0.005 | -0.151 |
|  | (0.003) | (0.101) |
| Number of observations | 5492 | 5492 |
| R2 | 0.0004 | 0.0004 |
| *Note: This table displays the effect of spending more time reading the texts on the ATEs of being allocated to the treatment or control groups on PEB1. We fitted OLS regressions to estimate the interaction between allocation in one of the treated or control groups and the time spent reading the articles. Robust standard errors are in parentheses. We apply Benjamini and Hochberg^61^ correction to conventional p-values (p). * p < 0.1, ** p < 0.05, *** p < 0.01.* p < 0.1, ** p < 0.05, *** p < 0.01.* | | |

Table C.4: Correlation between Perception of Effort and Information Treatments

| Data included | Participants who did PEB1 in wave 1 |
| --- | --- |
| Outcome | Effort |
|  | (1) |
| Constant | 2.441*** |
|  | (0.035) |
| All vs. Placebo | 0.230*** |
|  | (0.040) |
| Number of observations | 3806 |
| R2 | 0.009 |
| *Note: This table displays the effect of being shown information on PEB1 on the perception of having made an effort for the environment when participating in PEB1. Robust standard errors are in parentheses. We apply Benjamini and Hochberg^61^ correction to conventional p-values (p). * p < 0.1, ** p < 0.05, *** p < 0.01.* p < 0.1, ** p < 0.05, *** p < 0.01.* | |

***Appendix D. Randomization trees.***


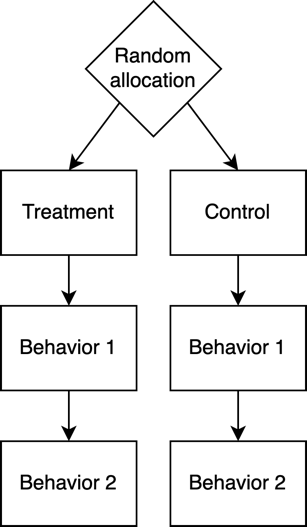


Figure 14: Randomization tree corresponding to designs of category 1

##
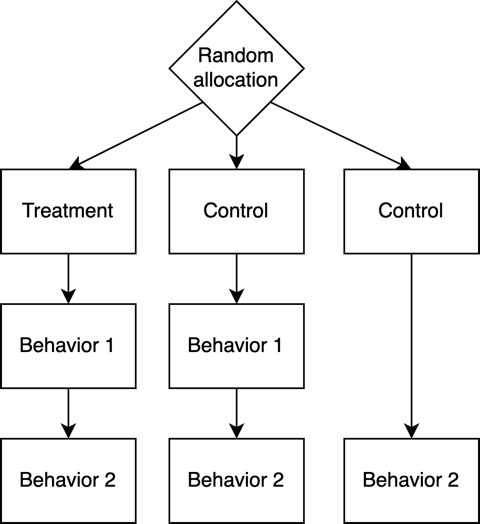


Figure 15: Randomization tree corresponding to designs of category 2


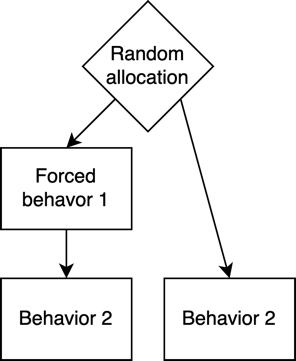


Figure 16: Randomization tree corresponding to designs of category 3


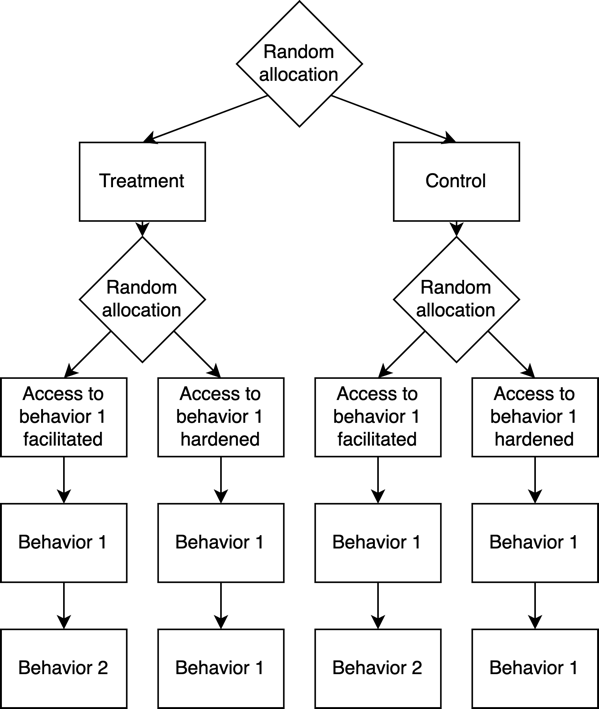


Figure 17: Randomization tree corresponding to the design we propose
